# Supplementary material for: The phosphatidylserine targeting antibody bavituximab plus pembrolizumab in unresectable hepatocellular carcinoma: a phase 2 trial
Source: Nat Commun. 2024 Mar 11;15:2178. doi: 10.1038/s41467-024-46542-y (PMC10928173; doi:10.1038/s41467-024-46542-y)
Supplement: Supplementary file 1 — Supplementary Information [file 41467_2024_46542_MOESM1_ESM.pdf]

Supplementary Table 1. Characteristics of evaluable patients.

Age, median, y (IQR)		64 (60-67)
Sex, no. (%)	Female	4 (14.3)
	Male	24 (85.7)
Race, no. (%)	Asian	0 (0)
	Black	14 (50)
	White	14 (50)
Ethnicity, no. (%)	Hispanic	2 (7.1)
	Non-Hispanic	26 (92.9)
Histology	Well	5 (17.9)
	Moderate	12 (42.9)
	Poor	6 (21.3)
	Unknown	5 (17.9)
Macrovascular invasion, no. (%)	Yes	8 (28.6)
	No	20 (71.4)
Etiology, no. (%)	HCV	20 (71.4)
	HBV	1 (3.6)
	EtOH	3 (10.7)
	NASH	4 (14.3)
Extrahepatic disease	Yes	14 (50.0)
	No	14 (50.0)
BCLC stage	B	7 (25.0)
	C	21 (75.0)
TNM stage	2	1 (3.6)
	3a	7 (25.0)
	3b	6 (21.3)
	4a	2 (7.1)
	4b	12 (42.9)
Prior locoregional therapy	Yes	17 (60.7)
	No	11 (39.3)
AFP	<400	16 (57.1)
	≥400	12 (42.9)

Supplementary Table 2. Tumor response in patients stratified by an RNA-based biomarker.

Best Overall Response N (%)	Evaluable patients[a] N =28	Patients with RNA-biomarker data[b]		
		All N =19	Immune high N = 8	Immune low N =11
Complete response	2 (7.1)	1 (5.3)	1 (12.5)	0 (0)
Partial response	7 (25.0)	5 (26.3)	4 (50.0)	1 (9.1)
Stable disease	5 (17.9)	3 (15.8)	1 (12.5)	2 (18.2)
Progressive disease	14 (50.0)	10 (52.6)	2 (25.0)	8 (72.7)
Objective response rate	9 (32.1)	6 (31.6)	5 (62.5)	1 (9.1)
Disease control rate	14 (50.0)	9 (47.4)	6 (75.0)	3 (27.3)

[a]Analysis based on evaluable patients per protocol.

[b]All patients with available biomarker results. 9 patients did not have tissue available for testing and biomarker status could not be determined.

Supplementary Figure 1. CONSORT diagram.

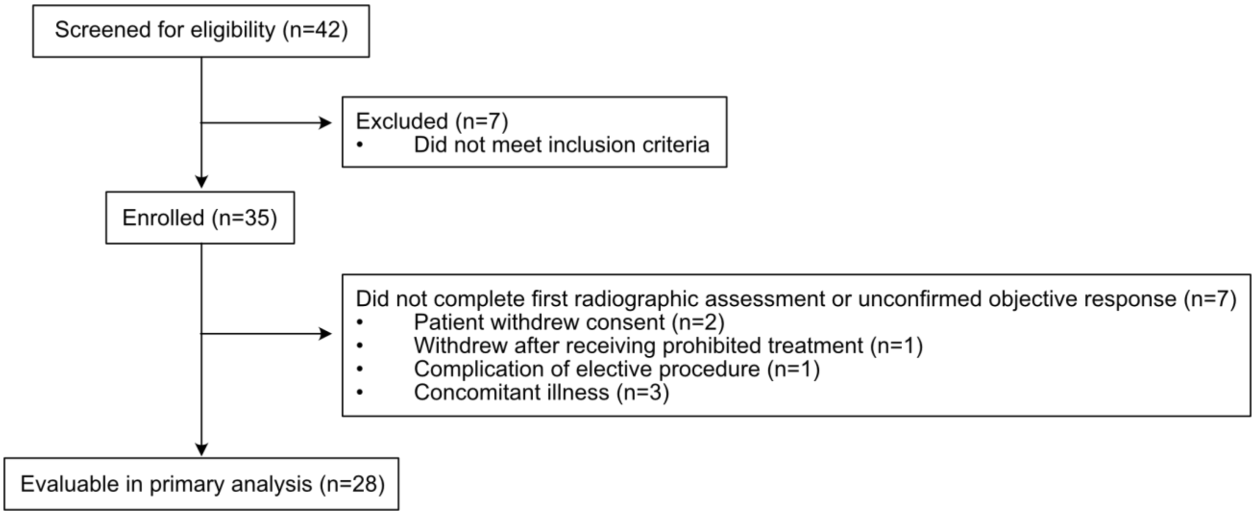

Supplementary Figure 2. Subgroup analyses of tumor response to bavituximab plus pembrolizumab.

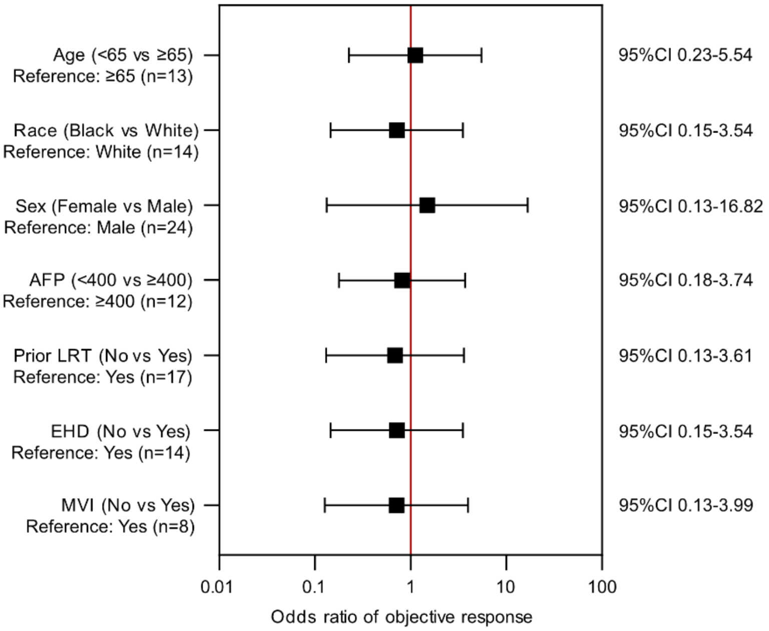

Forest plot of subgroup analyses of objective response by baseline demographics and disease characteristics. Odds ratios with 95% confidence intervals are from unadjusted analyses.

Supplementary Figure 3. Digital spatial profiling of immuno-oncology-related proteins in HCC tissues from patients treated with bavituximab and pembrolizumab.

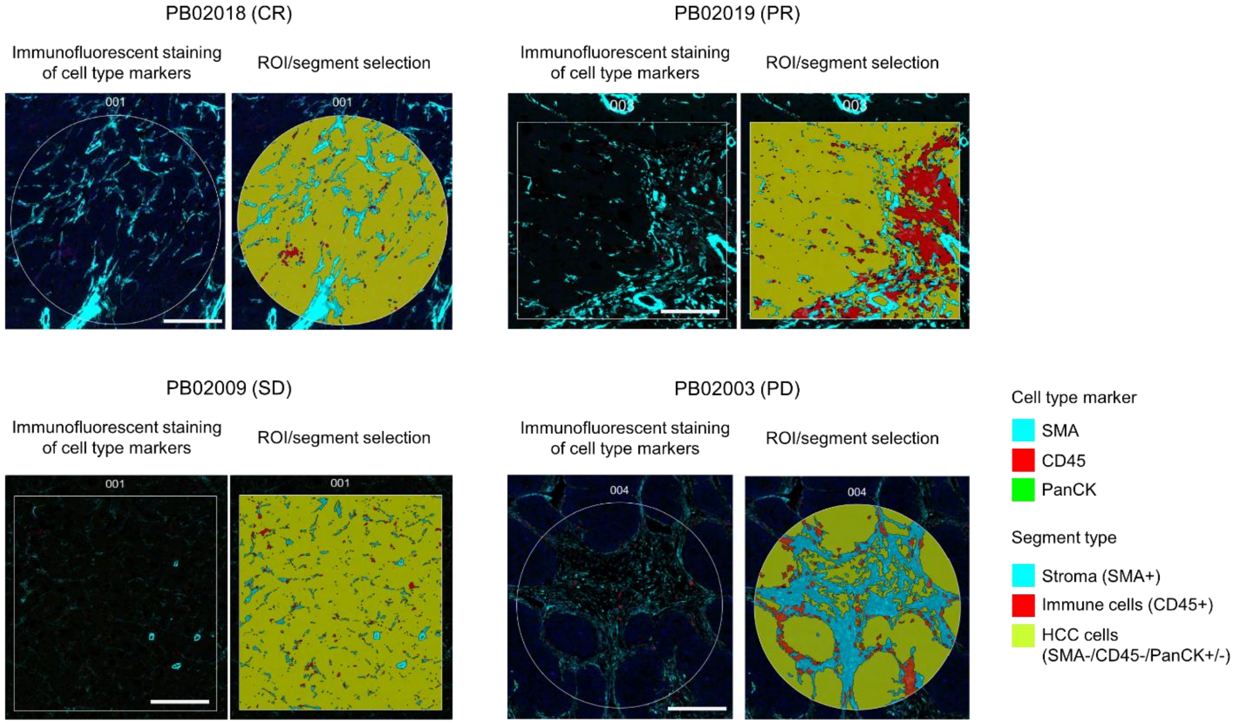

Representative images are shown demonstrating how stroma-, immune cell-, and HCC cell-enriched area (called "segment") in regions of interest (ROI) are defined by cell type markers alpha-smooth muscle actin (SMA), CD45, and pan-cytokeratin (or negativity of all markers with cancer cell morphology in H&E staining of serial tissue section), respectively. CR, complete response; PR, partial response; SD, stable disease; PD, progressive disease. Scale bar, 200 µm.

A Phase II Study of Pembrolizumab and Bavituximab in Patients with Advanced Hepatocellular Carcinoma

Principal Investigator:

David Hsieh, MD

Assistant Professor of Hematology Oncology
University of Texas Southwestern Medical Center
Harold C. Simmons Comprehensive Cancer Center
5323 Harry Hines Boulevard
Dallas, TX 75390-9179
Ph: 214-648-1996
Fx: 214-645-2542
Email: David.hsieh@utsouthwestern.edu

Co-Principal Investigators or Lead Sub-Investigator(s):

Adam Yopp, MD

Assistant Professor of Surgery

University of Texas Southwestern Medical Center

Harold C. Simmons Comprehensive Cancer Center

5323 Harry Hines Boulevard

Dallas, TX 75390-8852

Ph: 214-648-5870

Fx: 214-648-1118

Email: adam.yopp@utsouthwestern.edu

Biostatistician:

Hong Zhu, Ph.D.

UT Southwestern Medical Center

5323 Harry Hines Blvd

Dallas, TX 75390

NCT Number: 03519997

Amended: v6.0 24-August-2021

UT Southwestern Medical Center (UTSW)

Harold C. Simmons Comprehensive Cancer Center

Attn: Clinical Research Office

5323 Harry Hines Blvd. MC 9179

Dallas, Texas 75390-9179

Signature Page

The signature below constitutes the approval of this protocol and the attachments, and provides the necessary assurances that this trial will be conducted according to all stipulations of the protocol, including all statements regarding confidentiality, and according to local legal and regulatory requirements and applicable U.S. federal regulations and ICH guidelines.

Amendment/Version #______________________________

A Phase II Study of Pembrolizumab and Bavituximab in Patients with Advanced Hepatocellular Carcinoma

Principal Investigator (PI) Name: ______________________________

PI Signature: ______________________________

Date: ____________________

TABLE OF CONTENTS

LIST OF ABBREVIATIONS (EXAMPLES) **1**

STUDY SCHEMA **2**

STUDY SUMMARY **3**

1.0 BACKGROUND AND RATIONALE **5**

1.1 HEPATOCELLULAR DISEASE BACKGROUND 5

1.2 HEPATOCELLULAR CARCINOMA MANAGEMENT 5

1.3 DOSE RATIONALE 6

INTRODUCTION OF INVESTIGATIONAL TREATMENT(S) AND OTHER STUDY 9

1.4 TREATMENT(S) 9

1.5 STUDY RATIONALE 23

1.6 RATIONALE FOR TREATMENT BEYOND PROGRESSION 23

1.7 RATIONALE FOR EFFICACY ENDPOINTS 24

1.8 RESEARCH HYPOTHESIS 24

2.0 STUDY OBJECTIVES **24**

2.1 PRIMARY OBJECTIVE 24

2.2 SECONDARY OBJECTIVES 25

2.3 EXPLORATORY OBJECTIVES 25

3.0 STUDY DESIGN **25**

3.1 DESIGN 25

3.2 ENDPOINTS AND SAMPLE SIZE JUSTIFICATION 27

4.0 SUBJECT ELIGIBILITY **27**

4.1 INCLUSION CRITERIA 27

4.2 EXCLUSION CRITERIA 30

5.0 TREATMENT PLAN 31

5.1 OVERVIEW 31.2021

5.2 TREATMENT REGIMEN SCHEDULE AND DOSE ADJUSTMENTS 32

5.3 THERAPEUTIC/DIAGNOSTIC AGENTS 32

5.4 MONITORING OF STUDY TREATMENT DOSE ADMINISTRATION 34

5.5 TREATMENT DURATION 34

5.6 CONCOMITANT MEDICATIONS-ALLOWED AND PROHIBITED 35

5.7 DOSE MODIFICATION GUIDELINES 36

Table 5.2 Dose Modification Guidelines for Drug-Related Adverse Events 37

5.8 RESCUE MEDICATION AND SUPPORTIVE CARE 40

Diagnosis and Management of Non-Overdose Hepatic ECIs 46

5.9 CONTRACEPTION 49

5.10 DRUG SUPPLY AND STORAGE 51

6.0 STUDY PROCEDURES 51

6.1 SCREENING PHASE 51

6.2 TREATMENT PHASE 52

6.3 POST – TREATMENT FOLLOW-UP PHASE 53

7.0 MEASUREMENT OF EFFECT 1

7.1 ANTITUMOR EFFECT- SOLID TUMORS 1

7.2 TREATMENT BEYOND PROGRESSION 4

7.3 SAFETY/TOLERABILITY 5

8.0 ADVERSE EVENTS 5

8.1 PEMBROLIZUMAB TREATMENT RELATED EVENTS 5

8.2 BAVITUXIMAB TREATMENT RELATED EVENTS 6

8.3 ADVERSE EVENT MONITORING 6

8.4 STOPPING RULES 12

8.5 STEPS TO DETERMINE IF AN ADVERSE EVENT REQUIRES EXPEDITED REPORTING 12

8.6 DEFINITION OF AN OVERDOSE FOR THIS PROTOCOL AND REPORTING OF OVERDOSE TO MERCK 13

8.7 REPORTING OF PREGNANCY AND LACTATION TO MERCK 13

8.8 EVENTS OF CLINICAL INTEREST 13

9.0 CORRELATIVES/SPECIAL STUDIES 14

10.0 STUDY MANAGEMENT 15

10.1 CONFLICT OF INTEREST 15

10.2 INSTITUTIONAL REVIEW BOARD (IRB) APPROVAL AND CONSENT 15

10.3 RECRUITMENT PLAN 15

10.4 STUDY SUBJECT IDENTIFICATION NUMBER 16

10.5 SUBJECT REGISTRATION 16

10.6 PROTECTION OF HUMAN SUBJECTS 16

10.7 DATA MANAGEMENT AND MONITORING/AUDITING 17

10.8 ADHERENCE TO THE PROTOCOL 18

10.9 AMENDMENTS TO THE PROTOCOL 19

10.10 RECORD RETENTION 20

10.11 OBLIGATIONS OF INVESTIGATORS 20

11.0 REFERENCES 20

12.0 APPENDICES 24

Appendix A: ECOG Performance Status 24

Appendix B: Child-Pugh Score 24

Appendix C: NYHA Classification 25

LIST OF ABBREVIATIONS (EXAMPLES)

AE	Adverse Event
ALT	Alanine Aminotransferase
ALC	Absolute Lymphocyte Count
AST	Aspartate Aminotransferase
BUN	Blood Urea Nitrogen
CBC	Complete Blood Count
CMP	Comprehensive Metabolic Panel
CR	Complete Response
CT	Computed Tomography
CTCAE	Common Terminology Criteria for Adverse Events
DLT	Dose Limiting Toxicity
DSMB	Data and Safety Monitoring Board
ECOG	Eastern Cooperative Oncology Group
H&P	History and Physical Exam
HRPP	Human Research Protections Program
IV	Intravenously
mAB	Monoclonal antibody
MTD	Maximum Tolerated Dose
NCI	National Cancer Institute
ORR	Overall Response Rate
OS	Overall Survival
PBMCs	Peripheral Blood Mononuclear Cells
PD	Progressive Disease
PFS	Progression Free Survival
p.o.	Per os
PR	Partial Response
SAE	Serious Adverse Event
SD	Stable Disease
WBC	White Blood Count

STUDY SCHEMA

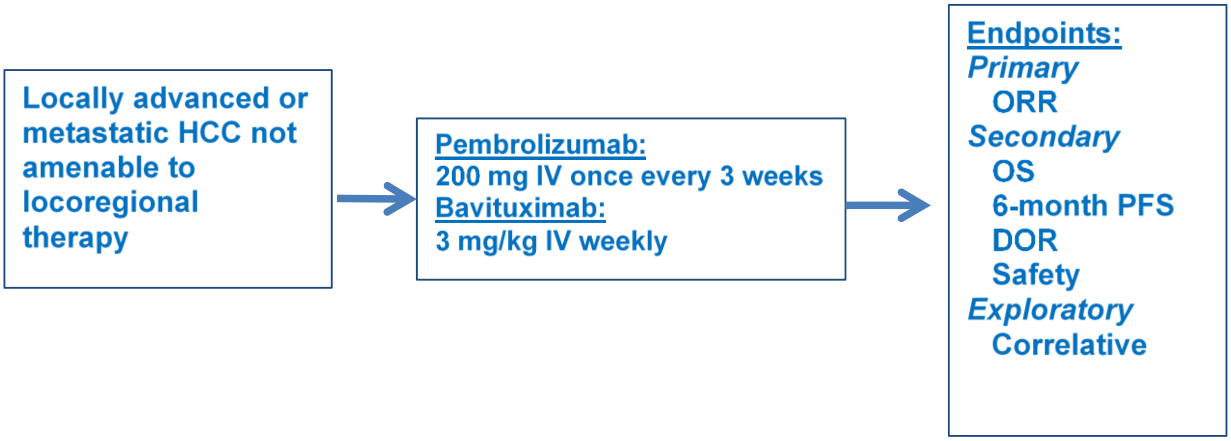

STUDY SUMMARY

Title	A Phase II Study of Pembrolizumab and Bavituximab In Patients With Advanced Hepatocellular Carcinoma (HCC)
Phase	Phase II
Methodology	Open label
Study Duration	24 months accrual/12 months follow-up
Study Center(s)	UT Southwestern Medical Center (UTSW) and<br>Parkland Health and Hospital System (PHHS)
Objectives	Primary objective:<br>**1.** To determine the overall response rate (ORR) of combination pembrolizumab and bavituximab in patients with advanced HCC.<br><br>Secondary objectives:<br>**1.** To determine overall survival, 6 month PFS, and duration of response of combination pembrolizumab and bavituximab compared to historical controls.<br>**2.** To determine the safety and tolerability of combination pembrolizumab and bavituximab as measured by rates of adverse events according to the CTCAE v. 4.03.<br>Exploratory objectives:<br>**1.** To determine treatment response with pre- and intra-treatment biopsies and plasma/serum correlative studies.
Number of Subjects	10-28
Diagnosis and Main Inclusion Criteria	1. Patients must have a histologically confirmed diagnosis hepatocellular carcinoma;<br>2. At least 4 weeks since prior therapy;<br>3. No history of prior systemic therapy for HCC;<br>4. Patients with advanced HCC not eligible for curative and/or locoregional therapies;<br>5. Age ≥ 18 years;<br>6. Child-Pugh Score A
Study Product(s), Dose, Route, Regimen	Pembrolizumab: 200 mg IV every 3 weeks Bavituximab:<br>3 mg/kg IV weekly
Duration of administration	Study treatment will continue until disease progression, unacceptable toxicity, death or discontinuation from the study treatment for any other reason. Subjects will be allowed to continue study therapy after an initial investigator-assessed RECIST 1.1-defined progression as long as the following criteria are met:<br>• Investigator assessed clinical benefit, and

	• Subject is tolerating the study drug (pembrolizumab and bavituximab).
Statistical Methodology	The primary endpoint of the trial will be overall response rate (ORR) of HCC patients. A minimax two-stage method will be used to analyze ORR after the first 15 patients are accrued. 13 additional patients will be enrolled if 3 or more of the first 15 patients have either a complete or partial response. ORR will be analyzed by RECIST 1.1 criteria.

1.0 BACKGROUND **AND RATIONALE**

1.1 HEPATOCELLULAR DISEASE BACKGROUND

Hepatocellular carcinoma (HCC) is the fifth most common cancer worldwide and is responsible for more than 500,000 deaths annually. (1) Previously considered uncommon in Western countries, the incidence and mortality of HCC have increased three-fold in the United States over the past two decades. (2)

1.2 HEPATOCELLULAR CARCINOMA MANAGEMENT

Although complete resection or hepatic transplantation remains the most effective therapies for localized HCC, 50-70% of patients present with advanced disease not amenable to curative surgical approaches. (3,4) Disease that is diagnosed at an advanced stage or with progression after locoregional therapy has a dismal prognosis with a median overall survival of < 12 months mainly due to underlying liver dysfunction and suboptimal treatment options. (2) Ablative therapies are commonly used in this setting but are ineffective for large (>5 cm) or multifocal tumors, both commonly found in advanced disease. (5, 6) Hepatic artery chemoembolization may have utility in patients with HCC but there are conflicting reports regarding its efficacy and in advanced disease a survival benefit has not been demonstrated. (7, 8)

Systemic Therapy for Advanced Hepatocellular Carcinoma

The use of single agent systemic cytotoxic chemotherapy regimens including irinotecan, gemcitabine or doxorubicin have historically demonstrated low response rates with little to no clinical efficacy. (9-11) Combination chemotherapy regimens using cytotoxic chemotherapeutic agents have fared no better with response rates ranging from 0% to 40% and limited clinical efficacy. A recent study compared single agent doxorubicin to a combination of cisplatin, interferon, doxorubicin and 5fluorouracil (PIAF). Despite an improvement in response (10% for doxorubicin vs. 21% for PIAF) the study failed to show an improvement in survival (6.8 vs. 8.6 months, respectively; P=0.83). (12)

The lack of appropriate traditional chemotherapeutic agents led to the investigation of molecular targeted agents, which have been shown to be efficacious in other tumor models. Sorafenib, an oral multikinase inhibitor that blocks tumor cell proliferation by targeting the Raf/MEK/ERK signaling pathway and exerts an antiangiogenic effect by targeting the tyrosine kinase receptors, VEGFR-2, VEGFR-3 and PDGF-□, is the first biologically targeted agent to show efficacy in the treatment of advanced stage HCC. (13, 14) In a phase III randomized controlled trial (SHARP trial), patients with advanced hepatocellular carcinoma (not eligible for surgical resection or transplantation) and preserved liver function (Child-Pugh A score) were randomly assigned to either systemic sorafenib or placebo. There was a significantly longer survival and radiologic time to progression (TTP) outcome in the cohort of patients that received sorafenib. Grade 3 drug related events included diarrhea (8% in the sorafenib group vs. 2% in the placebo group), hand-foot skin reaction (8% vs. 1%), hypertension (2% vs. 1%) and abdominal pain (2% vs. 1%); there were no grade 4 drug-related adverse events in any of these categories in either study group. Currently sorafenib is the only drug that has FDA approval for the systemic treatment of HCC. Although sorafenib is the current standard of care for patients with advanced HCC, it prolonged

radiologic time to progression less than 3 months when compared to best supportive care: 5.5 months versus 2.8 months, respectively. Overall survival was prolonged less than 3 months compared to best supportive care, 10.7 months versus 7.9 months, respectively. (14) Although systemic treatment with sorafenib shows statistically significant clinical efficacy, its overall benefit is modest.

Post-marketing clinical studies demonstrated that although sorafenib treatment has shown a significant increase in mean overall survival in different studies, only a portion of patients show real benefits, while the incidence of drug related significant adverse effects and the economic costs are relatively high. (15) Finally, predictive biomarkers for sorafenib benefit that could refine the risk-benefit ratio for therapy have not yet been validated. Although Regorafenib was approved by the FDA for treatment in a second line setting following Sorafenib in the first line setting,(16) there is a real need for treatment options for patients with advanced HCC.

1.3 DOSE RATIONALE

1.3.1 Rationale for Pembrolizumab in HCC

The liver has developed intrinsic mechanisms of immune tolerance to prevent abnormal immune responses to antigens. In HCC, this inherent anti-hepatic immunosuppressive environment is exacerbated by the chronic inflammation that underlies the development of fibrosis and cirrhosis. The altered environment results in oncogenic transformation and further activation of immunosuppressive mechanism, resulting in an inability to escalate a meaningful antitumor response. (17-19) Immune changes reported in HCC include tumor-associated antigen-specific CD8+ T-cell immune responses, T-cell infiltration after locoregional therapy, T regulatory cell (Treg) intratumoral accumulation, and myeloid-derived suppressor cells (MDSCs) accumulation. These changes have been correlated with disease progression and poor survival. (17)

There are reasons to believe that HCC is amenable to immunotherapy in that it has the highest reported rate of spontaneous regression, which is primarily attributed to antitumor immunity. Spontaneous regression of untreated lesions has been reported after locoregional treatment of liver tumors. (20, 21) These observations suggest that anti-tumor immune responses can be effective in HCC control.

There are data supporting a role for immunotherapy in HCC. Lymphocytic infiltration of the tumor and a high CD4+: CD8+ T-cell ratio has been associated with reduced risk of tumor recurrence following liver transplantation for HCC. (22) Adoptive immunotherapy with autologous lymphocytes activated with recombinant interleukin2 and antibody to CD3 in patients who had undergone curative resection resulted in a significantly longer recurrence-free and disease-specific survival, although overall survival did not differ significantly between treated and control groups. (23) The data nonetheless imply a

central role of T cells in modulating tumor progression and provide a strong justification for T cell immunotherapy. (24)

Preliminary data in HCC are available for inhibitors on the CTLA-4 and PD-1 checkpoint pathways. The CTLA-4 pathway inhibits T-cell activation, specifically Tregs. The phase II study of tremelimumab, a CTLA-4 blocking antibody, in HCC patients reported a partial response rate (PR) of 17.6%, a disease control rate (DCR) of 76.4%, and a time to progression of 6.5 months. (25) This compares favorably with sorafenib data (1% PR, 43% DCR, 5.5 months radiologic time to progression). (14)

A 2015 systemic review and meta-analysis investigated the potential value of PD-L1 in the prognostic prediction in human solid tumors. A total of 3107 patients with solid tumors from 28 published studies were included in the meta-analysis. The median percentage of solid tumors with PD-L1 overexpression was 53%. In this analysis, PD-L1 overexpression was associated with worse three-year OS in HCC. PD-L1 tumor staining of 40 commercial HCC tumor samples yielded positive staining in 13 of 40 (33%) including 45% of HCC samples without active hepatitis virus infection, 22% of HBV related HCC, and 0% with co-infected HCC. (26)

The phase I/II study of nivolumab, a PD-1 inhibitor, in HCC patients reported a 14% partial response rate, a 2% complete response rate, and an OS of 14.1 months.

Median duration of response was 17 months. Treatment-related serious adverse events was 7% with only 4% of patients discontinuing therapy due to adverse events. There were no treatment-related deaths. This compares a historical rate of 12-month OS after sorafenib failure of 30% and a grade 3 or 4 adverse event rate of 39% in sorafenib alone. (27)

Pembrolizumab is a potent humanized immunoglobulin G4 (IgG4) monoclonal antibody (mAb) with high specificity binding to the PD-1 receptor, thus inhibiting its interaction with PD-L1 and PD-L2. Based on preclinical, in vitro data, pembrolizumab has high affinity and potent receptor blocking activity for PD-1. The PD-1 checkpoint mediates the differentiation and proliferation of Tregs and also regulates peripheral tolerance and autoimmunity. The PD-1 pathway is activated binding of PD-1 to its ligands, PD-L1 and PD-L2, resulting in inhibition of T-cell proliferation and cytokine release. Animal data and translational clinical studies support a potential role of the PD-1/PD-L1 pathway as a mechanism for immune tolerance in HCC. PD-L1 is expressed by HCC cells and PD-1 is expressed by CD 8+ T cells in HCC tissue specimens. The level of expression significantly correlates with HCC stage, local recurrence rate, and poor prognosis. In addition, the frequency of intratumoral or circulating PD-1+ CD8+T cells correlates with HCC progression and postoperative recurrence.

Pembrolizumab is currently being investigated in multiple tumor types. Pembrolizumab treatment has demonstrated overall survival benefit in a phase 3 clinical trial in advanced melanoma and is currently approved for patients with nonsmall cell lung cancer with tumors expressing PD-L1. (28,29) In a trial of pembrolizumab versus ipilimumab in

advanced melanoma one-year survival was 74% (every 2 week dosing) and 68% (every 3 week dosing) for pembrolizumab and 58% for ipilimumab. The median progression free survival (PFS) was 5.5 months (every 2 week dosing) and 4.1 months (every 3 week dosing) for pembrolizumab and 2.8 months for ipilimumab. (28)

1.3.2 Rationale for Pembrolizumab Dose

The pembrolizumab dose of 200 mg every three weeks (Q3W) was selected based on clinical data and modeling and simulation approaches using population pharmacokinetics (PPK) and exposure-response analyses of data from studies in multiple tumor types (melanoma and NSCLC) where body weight normalized dosing (mg/kg) has been used.

PPK analyses have shown that the PK of pembrolizumab is linear with proportional exposure over a dose range of 1 to 10 mg/kg, and no differences in PK across ethnicities and tumor types were observed.

A population pharmacokinetic analysis has been performed using serum concentration time data from 476 patients. Within the resulting population PK model, clearance and volume parameters of pembrolizumab were found to be dependent on body weight. The relationship between clearance and body weight, with an allometric exponent of 0.59, is within the range observed for other antibodies and would support both body weight normalized dosing or a fixed dose across all body weights. Pembrolizumab has been found to have a wide therapeutic range based on the melanoma indication. The differences in exposure for a 200 mg fixed dose regimen relative to a 2 mg/kg Q3W body weight based regimen are anticipated to remain well within the established exposure margins of 0.5 – 5.0 for pembrolizumab in the melanoma indication. The exposure margins are based on the notion of similar efficacy and safety in melanoma at 10 mg/kg Q3W vs. the proposed dose regimen of 2 mg/kg Q3W (i.e. 5-fold higher dose and exposure). The population PK evaluation revealed that there was no significant impact of tumor burden on exposure. In addition, exposure was similar between the NSCLC and melanoma indications. Therefore, there are no anticipated changes in exposure between different indication settings.

The choice of the 200 mg Q3W as an appropriate dose for the switch to fixed dosing is based on simulations performed using the population PK model of pembrolizumab showing that the fixed dose of 200 mg every 3 weeks will provide exposures that 1) are optimally consistent with those obtained with the 2 mg/kg dose every 3 weeks, 2) will maintain individual patient exposures in the exposure range established in melanoma as associated with maximal efficacy response and 3) will maintain individual patients exposure in the exposure range established in melanoma that are well tolerated and safe.

A fixed dose regimen will simplify the dosing regimen to be more convenient for physicians and to reduce potential for dosing errors. A fixed dosing scheme will also reduce complexity in the logistical chain at treatment facilities and reduce wastage.

1.3.3 Rationale for Bavituximab in HCC

Bavituximab is a genetically engineered immunoglobulin gamma 1 (IgG1) chimeric (human/mouse) mAb containing the variable region sequences of the murine PStargeting mouse antibody 3G4(2aG4) and human IgG1 k constant region sequences that targets PS after binding to □2-glycoprotein 1 (□2GP1). PS is a highly immunosuppressive molecule typically expressed on the inner leaflet of the plasma membrane of normal cells but “flips” and becomes externalized to the outer leaflet of the plasma membrane on cells that line tumor blood vessels, tumor cells and exosomes in the tumor microenvironment creating a specific target for anticancer treatments.

Bavituximab is a novel immunotherapeutic approach because its primary mechanism of action involves modulating the tumor microenvironment from a primarily immunosuppressive, angiogenesis-promoting state (with infiltrating myeloid-derived suppressor cells [MDSCs] and M2-macrophages) to an immune activating state (with long-term tumor specific-immunity facilitated by M1 macrophages, mature dendritic cells, and activated T lymphocytes) that drives both innate and adaptive immunity. Bavituximab blocks the immunosuppressive signaling of myeloid derived cells and sends and alternate immune activation signal leading to an adaptive T-cell mediated anti-tumor response. In addition, through activation of innate immunity (i.e., M1 macrophages and natural killer [NK] cells), bavituximab causes tumor cell destruction and the selective shutdown of pre-existing tumor blood vessels expressing PS via antibody-dependent cell mediated cytotoxicity.

To date, the clinical development program of bavituximab has enrolled approximately 900 patients in oncology studies. Specifically a phase I/II clinical trial has demonstrated that radiological TTP with bavituximab combined with sorafenib was 6.7 months compared to the historical controls of 5.5 months from the SHARP trial with sorafenib alone. The 6-month PFS in the bavituximab and sorafenib combination group was 20%. The dosage of bavituximab in the phase I/II trial in HCC patients was 3.0 mg/kg weekly intravenously and not associated with any severe treatment related adverse events.(30)

1.4 INTRODUCTION OF INVESTIGATIONAL TREATMENT(S) AND OTHER STUDY TREATMENT(S)

1.4.1 Overview of pembrolizumab

Pembrolizumab is a potent and highly selective humanized monoclonal antibody (mAb) of the immunoglobulin G4 (IgG4)/kappa isotype designed to directly block the interaction between PD-1 and its ligands, programmed cell death ligand 1 (PDL1) and programmed cell death ligand 2 (PD-L2). This blockade enhances functional activity of the target lymphocytes to facilitate tumor regression and ultimately immune rejection.

1.4.2 Pembrolizumab non-clinical experience

Nonclinical Pharmacology

Pembrolizumab binds to human and Cynomolgus monkey PD-1 with comparable affinity and blocks the binding of human and Cynomolgus monkey PD-1 to PDL1 and PD-L2 with comparable potency. Pembrolizumab does not cross-react with dog, rat, or mouse PD-1. Pembrolizumab does not bind immunoglobulin superfamily members cluster of differentiation 28 (CD28), cytotoxic Tlymphocyte-associated protein 4 (CTLA-4), or inducible T-cell costimulator (ICOS).

Pembrolizumab strongly enhances T-lymphocyte immune responses in cultured blood cells from healthy human donors, cancer subjects, and nonhuman primates. In T-cell activation assays using human donor blood cells, the halfmaximal effective concentration (EC50) has been approximately 0.1 to 0.3 nM. In addition to interleukin-2 (IL-2), tumor necrosis factor alpha (TNFα), interferon gamma (IFN), and levels of other cytokines were found to be modulated by pembrolizumab. The antibody potentiates existing immune responses only in the presence of antigen and does not nonspecifically activate T-cells. In the in vitro peripheral blood mononuclear cell (PBMC) and whole blood cytokine release assays, the cytokine levels induced by pembrolizumab were low and comparable to those induced by trastuzumab. Pembrolizumab does not induce antibodydependent cell-mediated cytotoxicity (ADCC) or complement-dependent cytotoxicity (CDC).

Using anti–murine PD-1 surrogate antibodies, PD-1 blockade has been shown to significantly inhibit tumor growth in a variety of syngeneic murine tumor models. In these experiments in mice, anti–PD-1 therapy is synergistic with chemotherapeutic agents such as gemcitabine and 5-fluorouracil (5-FU), and combination therapy results in increased complete tumor regression rates in vivo. Studies also revealed that immunosuppressive doses of dexamethasone included in combination with agents used in standard-of-care treatment for NSCLC do not reduce the anti-tumor efficacy of an anti–murine PD-1 surrogate antibody.

Nonclinical Pharmacokinetics

The pharmacokinetics (PK) of pembrolizumab were evaluated in a non–Good Laboratory Practices (GLP) single dose PK study and two GLP repeat-dose toxicokinetic (TK) studies (1 month and 6 month) in Cynomolgus monkeys. Pembrolizumab stability as a modified IgG4 molecule was evaluated in vivo in mice.

After single-dose IV administration at 0.3, 3, or 30 mg/kg in Cynomolgus monkeys, decline of serum concentration followed multiphasic kinetics. Anti–drug antibodies (ADAs) were detected in most of the treated animals. Clearance (CL) and terminal half-life (t1/2) appeared to be dose-dependent in the dose range tested with t1/2 varying from 4 to 10 days.

In the 1-month repeat-dose (once weekly) GLP toxicity study at 6, 40, or 200 mg/kg in Cynomolgus monkeys, ADAs were detected in most of the low-dose (6 mg/kg) treated

animals. The systemic exposure over the 7-day dosing interval (AUC0-7 days) was sex-independent and increased with increasing dose. The mean t1/2 values in individual ADA-negative animals ranged from 15.7 to 22.3 days across doses.

In the 6-month repeat-dose (every other week) GLP toxicity study at 6, 40, or 200 mg/kg in Cynomolgus monkeys, ADAs were detected in most of the low-dose (6 mg/kg) treated animals. The systemic exposure to pembrolizumab was independent of sex and was approximately dose-proportional across all doses. The mean t1/2 values in individual ADA-negative animals ranged from 21 to 22 days across doses.

IgG4 wild type molecule can undergo in vivo molecular rearrangement called Fabarm (or half molecule) exchange by swapping their half molecule with other IgG4 half molecules, thereby generating bispecific or hybrid antibodies. Pembrolizumab is a hinge mutated IgG4 (S228P), which prevents in vivo half-molecule swap (formation of hybrid). An in vivo mice experiment has demonstrated that pembrolizumab did not form hybrid antibody with another wild type IgG4 molecule.

Safety Pharmacology/Toxicology

The potential for systemic toxicity of pembrolizumab was assessed in a 1-month repeat-dose toxicity study with a 4-month recovery in Cynomolgus monkeys and in a 6-month repeat-dose toxicity study with a 4-month recovery period in Cynomolgus monkeys. In the 1-month toxicity study, Cynomolgus monkeys were administered an IV dose of 6, 40, or 200 mg/kg once weekly for a total of 5 doses. Four monkeys/sex/group were euthanized during Week 5. The remaining 2 monkeys/sex/group were euthanized during Week 23, after a 4-month postdose period. In this study, pembrolizumab was well-tolerated in monkeys with the systemic exposure (AUC) up to approximately 170,000 µg·day/mL over the course of the study. There was no test article–related mortality, and test article–related changes were limited to an increased incidence of inguinal swelling, and increased splenic weights in males receiving 200 mg/kg. Both of these findings were not considered adverse and there was no histopathologic correlation. Splenic weights were normal at the postdose necropsy. Anti–pembrolizumab antibodies were detected in 7 out of 8 animals in the 6 mg/kg dose group and 1 animal out of 8 in the 40 mg/kg dose group and were associated with an apparent increase in clearance of pembrolizumab. The presence of ADA in monkeys in the low-dose group and in 1 monkey in the mid-dose group did not impact the pharmacodynamic response, because sufficient target engagement was demonstrated for the duration of the study (with the exception of 1 low-dose monkey). Additionally, anti–pembrolizumab antibodies were not detected in any monkeys in the highdose group, suggesting that potential toxicity has been evaluated at the highest exposure levels in the study. Based on the lack of adverse test article–related findings in this study, the no observed adverse effect level (NOAEL) was ≥200 mg/kg.

In the 6-month toxicity study, the potential for systemic toxicity was assessed in Cynomolgus monkeys administered an IV dose of 6, 40, or 200 mg/kg once every other week for approximately 6 months (a total of 12 doses) followed by a 4-month treatment-free period. Three animals/sex/group were designated for interim necropsy at the end of the 6-monthdosing phase (3 days after receiving the last dose in Study Week 23); and the remaining monkeys were designated for final necropsy following the 4-month treatment-free period. Pembrolizumab was well tolerated at all dose levels. There were no test

article–related antemortem findings, electrocardiographic or ophthalmic findings, changes at injection sites, gross observations or organ weight changes at the interim or final necropsy. Because there were no test article–related histomorphologic findings at interim necropsy, histomorphologic evaluation of tissues collected at final necropsy was not conducted. The presence of ADA was observed in 5 out of 10 animals at 6 mg/kg/dose during the dosing phase, which correlated with an apparent increased rate of elimination of pembrolizumab in these animals. No anti–pembrolizumab antibodies were detected at 40 or 200 mg/kg/dose during the dosing phase, and no pembrolizumab serum concentration profiles in these 2 groups suggested an effect of ADA on pembrolizumab elimination rate. During the treatment-free period, anti– pembrolizumab antibodies were detected in 2 animals at the 6 mg/kg/dose, which already had ADA present during the dosing phase, and in 2 additional animals (1 at the 6 mg/kg/dose and 1 at the 200 mg/kg/dose), which were ADA-negative during the dosing phase. The detection of anti-pembrolizumab antibodies had a minimal effect on the mean group systemic exposure to pembrolizumab during the study and did not impact the evaluation of potential toxicity of pembrolizumab for the duration of the 6-month study, because there were no test article–related effects on any of the parameters examined and no monkey in the mid- and high-dose groups developed ADA during the dosing phase. In conclusion, pembrolizumab administered once every other week over a 6-month duration to Cynomolgus monkeys was well tolerated and the NOAEL was ≥200 mg/kg/dose (the highest dose tested).

In addition, tissue cross-reactivity studies using monkey and human specimens were conducted to evaluate the potential cross reactivity of pembrolizumab with cryosections of Cynomolgus monkey tissues and normal human tissues. Results demonstrated the expected on-target staining of the membranes of mononuclear leukocytes in both species. The off-target staining (cytoplasmic and stromal) that occurred in many tissues of both species was considered spurious binding inherent to the experimental conditions of the in vitro tissue cross-reactivity studies with no in vivo toxicological significance.

Detailed information about the comprehensive nonclinical safety, immunology, and pharmacokinetic (PK) testing program is provided in the current version of the pembrolizumab Investigator's Brochure.

1.4.3 Pembrolizumab clinical experience

As of August 15, 2015, 1195 patients with melanoma have being treated in 2 clinical trials (P01 and P02). Patients in P01 were treated with 2 mg/kg every 3 weeks, 10 mg/kg every three weeks, or 10 mg/kg every 2 weeks. The primary endpoint was objective response rate (ORR) with disease control rate (DCR) a secondary endpoint. Overall there were 44 complete responses and 159 partial responses. The ORR was 31% (95% CI: 28% to 35%). DCR was achieved in 51% of all subjects. Patients in P02 were treated with 2 mg/kg every 3 weeks and 10 mg/kg every 3 weeks versus investigator choice chemotherapy in a 1:1:1 ratio in subjects with IPIrefractory melanoma. The two coprimary efficacy endpoints were PFS and OS. The median PFS was 2.9 months in both pembrolizumab arms and 2.7 months in the control arm. A preliminary analysis of OS indicated that the hazard ration was 0.88 in the pembrolizumab 2 mg/kg Q3W arm over the control arm and 0.78 in the pembrolizumab 10 mg/kg Q3W arm over the control arm. The one-sided p-value was 0.229 and 0.066 in 2 mg/kg Q3W and 10 mg/kg Q3W over the control arm, respectively, both favoring pembrolizumab.

The most common all-grade adverse events (>10%) for the P01 and P02 clinical trials are shown below.

	MK-3475 2 mg/kg Q3W		MK-3475 10 mg/kg Q3W		MK-3475 10 mg/kg Q2W		Total	
	n	(%)	n	(%)	n	(%)	n	(%)
Subjects in population	401		779		382		1,562	
with one or more adverse events	390	(97.3)	759	(97.4)	375	(98.2)	1,524	(97.6)
with no adverse events	11	(2.7)	20	(2.6)	7	(1.8)	38	(2.4)
**Blood and lymphatic system disorders**	**71**	**(17.7)**	**131**	**(16.8)**	**80**	**(20.9)**	**282**	**(18.1)**
Anaemia	61	(15.2)	94	(12.1)	60	(15.7)	215	(13.8)
**Cardiac disorders**	**32**	**(8.0)**	**63**	**(8.1)**	**40**	**(10.5)**	**135**	**(8.6)**
**Endocrine disorders**	**42**	**(10.5)**	**72**	**(9.2)**	**42**	**(11.0)**	**156**	**(10.0)**
**Eye disorders**	**57**	**(14.2)**	**94**	**(12.1)**	**45**	**(11.8)**	**196**	**(12.5)**
**Gastrointestinal disorders**	**240**	**(59.9)**	**482**	**(61.9)**	**240**	**(62.8)**	**962**	**(61.6)**
Abdominal pain	47	(11.7)	81	(10.4)	41	(10.7)	169	(10.8)
Constipation	82	(20.4)	139	(17.8)	69	(18.1)	290	(18.6)
Diarrhoea	87	(21.7)	174	(22.3)	89	(23.3)	350	(22.4)
Nausea	91	(22.7)	190	(24.4)	105	(27.5)	386	(24.7)
Vomiting	49	(12.2)	114	(14.6)	58	(15.2)	221	(14.1)
**General disorders and administration site conditions**	**254**	**(63.3)**	**544**	**(69.8)**	**285**	**(74.6)**	**1,083**	**(69.3)**
Asthenia	42	(10.5)	88	(11.3)	49	(12.8)	179	(11.5)
Fatigue	151	(37.7)	339	(43.5)	172	(45.0)	662	(42.4)
Oedema peripheral	42	(10.5)	87	(11.2)	47	(12.3)	176	(11.3)
Pyrexia	46	(11.5)	106	(13.6)	58	(15.2)	210	(13.4)
**Infections and infestations**	**161**	**(40.1)**	**291**	**(37.4)**	**154**	**(40.3)**	**606**	**(38.8)**
**Injury, poisoning and procedural complications**	**61**	**(15.2)**	**89**	**(11.4)**	**45**	**(11.8)**	**195**	**(12.5)**
**Investigations**	**125**	**(31.2)**	**226**	**(29.0)**	**116**	**(30.4)**	**467**	**(29.9)**
**Metabolism and nutrition disorders**	**141**	**(35.2)**	**318**	**(40.8)**	**170**	**(44.5)**	**629**	**(40.3)**

	MK-3475 2 mg/kg Q3W		MK-3475 10 mg/kg Q3W		MK-3475 10 mg/kg Q2W		Total	
	n	(%)	n	(%)	n	(%)	n	(%)
**Metabolism and nutrition disorders**	**141**	**(35.2)**	**318**	**(40.8)**	**170**	**(44.5)**	**629**	**(40.3)**
Decreased appetite	70	(17.5)	183	(23.5)	95	(24.9)	348	(22.3)
**Musculoskeletal and connective tissue disorders**	**194**	**(48.4)**	**384**	**(49.3)**	**203**	**(53.1)**	**781**	**(50.0)**
Arthralgia	72	(18.0)	143	(18.4)	85	(22.3)	300	(19.2)
Back pain	45	(11.2)	84	(10.8)	48	(12.6)	177	(11.3)
Myalgia	35	(8.7)	64	(8.2)	52	(13.6)	151	(9.7)
Pain in extremity	41	(10.2)	57	(7.3)	35	(9.2)	133	(8.5)
**Nervous system disorders**	**150**	**(37.4)**	**289**	**(37.1)**	**143**	**(37.4)**	**582**	**(37.3)**
Headache	46	(11.5)	126	(16.2)	60	(15.7)	232	(14.9)
**Psychiatric disorders**	**71**	**(17.7)**	**140**	**(18.0)**	**79**	**(20.7)**	**290**	**(18.6)**
**Renal and urinary disorders**	**35**	**(8.7)**	**72**	**(9.2)**	**50**	**(13.1)**	**157**	**(10.1)**
**Respiratory, thoracic and mediastinal disorders**	**172**	**(42.9)**	**389**	**(49.9)**	**228**	**(59.7)**	**789**	**(50.5)**
Cough	85	(21.2)	183	(23.5)	93	(24.3)	361	(23.1)
Dyspnoea	59	(14.7)	157	(20.2)	84	(22.0)	300	(19.2)
**Skin and subcutaneous tissue disorders**	**195**	**(48.6)**	**403**	**(51.7)**	**174**	**(45.5)**	**772**	**(49.4)**
Pruritus	90	(22.4)	185	(23.7)	78	(20.4)	353	(22.6)
Rash	69	(17.2)	143	(18.4)	62	(16.2)	274	(17.5)
**Vascular disorders**	**40**	**(10.0)**	**110**	**(14.1)**	**62**	**(16.2)**	**212**	**(13.6)**

Every subject is counted a single time for each applicable row and column.

A system organ class or specific adverse event appears on this report only if its incidence in one or more of the columns meets the incidence criterion in the report title, after rounding.

MedDRA preferred terms "Neoplasm Progression," "Malignant Neoplasm Progression," and "Disease Progression" not related to the drug are excluded.

Include all treated subjects in P001 Parts B1, B2, B3, D, C, F1, F2, and F3 and all subjects in P002 treated with pembrolizumab in the original phase.

(MK-3475 P001 Database Cutoff Date for Melanoma: 18APR2014).

(MK-3475 P001 Database Cutoff Date for Lung: 29AUG2014).

(MK-3475 P002 Database Cutoff Date: 12MAY2014).

The most common grade 3-5 adverse events in P01 and P02 clinical trials are shown below.

	MK-3475 2 mg/kg Q3W		MK-375 10 mg/kg Q3W		MK-3475 10 mg/kg Q2W		Total	
	n	(%)	n	(%)	n	(%)	n	(%)
Subjects in population	401		779		382		1,562	
with one or more adverse events	53	(13.2)	83	(10.7)	51	(13.4)	187	(12.0)
with no adverse events	348	(86.8)	696	(89.3)	331	(86.6)	1,375	(88.0)
**Blood and lymphatic system disorders**	**5**	**(1.2)**	**3**	**(0.4)**	**9**	**(2.4)**	**17**	**(1.1)**
Anaemia	4	(1.0)	1	(0.1)	2	(0.5)	7	(0.4)
Autoimmune haemolytic anaemia	0	(0.0)	0	(0.0)	1	(0.3)	1	(0.1)
Haemolytic anaemia	0	(0.0)	0	(0.0)	1	(0.3)	1	(0.1)
Immune thrombocytopenic purpura	0	(0.0)	0	(0.0)	1	(0.3)	1	(0.1)
Leukopenia	0	(0.0)	1	(0.1)	1	(0.3)	2	(0.1)
Lymphopenia	0	(0.0)	0	(0.0)	1	(0.3)	1	(0.1)
Neutropenia	1	(0.2)	0	(0.0)	1	(0.3)	2	(0.1)
Pancytopenia	0	(0.0)	0	(0.0)	1	(0.3)	1	(0.1)
Thrombocytopenia	0	(0.0)	1	(0.1)	0	(0.0)	1	(0.1)
**Cardiac disorders**	**3**	**(0.7)**	**3**	**(0.4)**	**0**	**(0.0)**	**6**	**(0.4)**
Acute myocardial infarction	0	(0.0)	1	(0.1)	0	(0.0)	1	(0.1)
Cardiac tamponade	0	(0.0)	1	(0.1)	0	(0.0)	1	(0.1)
Cardio-respiratory arrest	1	(0.2)	0	(0.0)	0	(0.0)	1	(0.1)
Pericardial effusion	2	(0.5)	1	(0.1)	0	(0.0)	3	(0.2)
Pericarditis	1	(0.2)	0	(0.0)	0	(0.0)	1	(0.1)
**Endocrine disorders**	**2**	**(0.5)**	**8**	**(1.0)**	**2**	**(0.5)**	**12**	**(0.8)**
Adrenal insufficiency	0	(0.0)	3	(0.4)	0	(0.0)	3	(0.2)
Hyperthyroidism	0	(0.0)	2	(0.3)	1	(0.3)	3	(0.2)
Hypophysitis	2	(0.5)	1	(0.1)	0	(0.0)	3	(0.2)
Hypopituitarism	0	(0.0)	2	(0.3)	0	(0.0)	2	(0.1)
Hypothyroidism	0	(0.0)	1	(0.1)	1	(0.3)	2	(0.1)
**Eye disorders**	**0**	**(0.0)**	**2**	**(0.3)**	**0**	**(0.0)**	**2**	**(0.1)**
Eye pain	0	(0.0)	1	(0.1)	0	(0.0)	1	(0.1)
Iritis	0	(0.0)	1	(0.1)	0	(0.0)	1	(0.1)
**Gastrointestinal disorders**	**11**	**(2.7)**	**16**	**(2.1)**	**9**	**(2.4)**	**36**	**(2.3)**

	MK-3475 2 mg/kg Q3W		MK-3475 10 mg/kg Q3W		MK-3475 10 mg/kg Q2W		Total	
	n	(%)	n	(%)	n	(%)	n	(%)
**Gastrointestinal disorders**	**11**	**(2.7)**	**16**	**(2.1)**	**9**	**(2.4)**	**36**	**(2.3)**
Abdominal pain	0	(0.0)	1	(0.1)	0	(0.0)	1	(0.1)
Ascites	1	(0.2)	0	(0.0)	0	(0.0)	1	(0.1)
Colitis	3	(0.7)	5	(0.6)	1	(0.3)	9	(0.6)
Diarrhoea	1	(0.2)	6	(0.8)	3	(0.8)	10	(0.6)
Dysphagia	1	(0.2)	0	(0.0)	0	(0.0)	1	(0.1)
Enterocolitis	0	(0.0)	0	(0.0)	1	(0.3)	1	(0.1)
Intestinal obstruction	0	(0.0)	0	(0.0)	1	(0.3)	1	(0.1)
Intussusception	0	(0.0)	0	(0.0)	1	(0.3)	1	(0.1)
Nausea	1	(0.2)	2	(0.3)	2	(0.5)	5	(0.3)
Oral pain	0	(0.0)	1	(0.1)	0	(0.0)	1	(0.1)
Pancreatitis	0	(0.0)	1	(0.1)	0	(0.0)	1	(0.1)
Rectal haemorrhage	0	(0.0)	0	(0.0)	1	(0.3)	1	(0.1)
Small intestinal perforation	1	(0.2)	0	(0.0)	0	(0.0)	1	(0.1)
Upper gastrointestinal haemorrhage	1	(0.2)	0	(0.0)	0	(0.0)	1	(0.1)
Vomiting	3	(0.7)	2	(0.3)	1	(0.3)	6	(0.4)
**General disorders and administration site conditions**	**14**	**(3.5)**	**11**	**(1.4)**	**6**	**(1.6)**	**31**	**(2.0)**
Asthenia	3	(0.7)	3	(0.4)	1	(0.3)	7	(0.4)
Chest pain	1	(0.2)	0	(0.0)	0	(0.0)	1	(0.1)
Death	1	(0.2)	0	(0.0)	0	(0.0)	1	(0.1)
Fatigue	8	(2.0)	5	(0.6)	5	(1.3)	18	(1.2)
Generalised oedema	2	(0.5)	1	(0.1)	0	(0.0)	3	(0.2)
Mucosal inflammation	0	(0.0)	1	(0.1)	0	(0.0)	1	(0.1)
Pain	0	(0.0)	0	(0.0)	1	(0.3)	1	(0.1)
Pyrexia	0	(0.0)	1	(0.1)	0	(0.0)	1	(0.1)
**Hepatobiliary disorders**	**3**	**(0.7)**	**2**	**(0.3)**	**1**	**(0.3)**	**6**	**(0.4)**
Autoimmune hepatitis	3	(0.7)	1	(0.1)	0	(0.0)	4	(0.3)
Hepatitis	0	(0.0)	1	(0.1)	0	(0.0)	1	(0.1)
Hyperbilirubinaemia	0	(0.0)	0	(0.0)	1	(0.3)	1	(0.1)
**Immune system disorders**	**0**	**(0.0)**	**2**	**(0.3)**	**0**	**(0.0)**	**2**	**(0.1)**
Anaphylactic reaction	0	(0.0)	1	(0.1)	0	(0.0)	1	(0.1)
Serum sickness	0	(0.0)	1	(0.1)	0	(0.0)	1	(0.1)

	MK-3475 2 mg/kg Q3W		MK-3475 10 mg/kg Q3W		MK-3475 10 mg/kg Q2W		Total	
	n	(%)	n	(%)	n	(%)	n	(%)
**Infections and infestations**	**0**	**(0.0)**	**3**	**(0.4)**	**2**	**(0.5)**	**5**	**(0.3)**
Clostridium difficile infection	0	(0.0)	1	(0.1)	0	(0.0)	1	(0.1)
Diverticulitis	0	(0.0)	1	(0.1)	0	(0.0)	1	(0.1)
Erysipelas	0	(0.0)	1	(0.1)	0	(0.0)	1	(0.1)
Meningitis	0	(0.0)	0	(0.0)	1	(0.3)	1	(0.1)
Pneumonia	0	(0.0)	0	(0.0)	1	(0.3)	1	(0.1)
**Investigations**	**5**	**(1.2)**	**10**	**(1.3)**	**5**	**(1.3)**	**20**	**(1.3)**
Alanine aminotransferase increased	0	(0.0)	3	(0.4)	1	(0.3)	4	(0.3)
Amylase increased	1	(0.2)	0	(0.0)	0	(0.0)	1	(0.1)
Aspartate aminotransferase increased	1	(0.2)	3	(0.4)	1	(0.3)	5	(0.3)
Blood alkaline phosphatase increased	0	(0.0)	1	(0.1)	0	(0.0)	1	(0.1)
Blood bilirubin increased	0	(0.0)	1	(0.1)	0	(0.0)	1	(0.1)
Blood creatine phosphokinase increased	1	(0.2)	1	(0.1)	1	(0.3)	3	(0.2)
Blood glucose increased	0	(0.0)	1	(0.1)	0	(0.0)	1	(0.1)
Gamma-glutamyltransferase increased	2	(0.5)	1	(0.1)	1	(0.3)	4	(0.3)
Lymphocyte count decreased	1	(0.2)	1	(0.1)	0	(0.0)	2	(0.1)
Neutrophil count decreased	0	(0.0)	0	(0.0)	1	(0.3)	1	(0.1)
Transaminases increased	0	(0.0)	1	(0.1)	0	(0.0)	1	(0.1)
Weight decreased	0	(0.0)	1	(0.1)	1	(0.3)	2	(0.1)
**Metabolism and nutrition disorders**	**3**	**(0.7)**	**9**	**(1.2)**	**7**	**(1.8)**	**19**	**(1.2)**
Decreased appetite	0	(0.0)	3	(0.4)	1	(0.3)	4	(0.3)
Dehydration	1	(0.2)	1	(0.1)	1	(0.3)	3	(0.2)

	MK-3475 2 mg/kg Q3W		MK-3475 10 mg/kg Q3W		MK-3475 10 mg/kg Q2W		Total	
	n	(%)	n	(%)	n	(%)	n	(%)
**Metabolism and nutrition disorders**	**3**	**(0.7)**	**9**	**(1.2)**	**7**	**(1.8)**	**19**	**(1.2)**
Failure to thrive	0	(0.0)	0	(0.0)	1	(0.3)	1	(0.1)
Hyperglycaemia	0	(0.0)	1	(0.1)	1	(0.3)	2	(0.1)
Hypertriglyceridaemia	0	(0.0)	0	(0.0)	1	(0.3)	1	(0.1)
Hypoalbuminaemia	1	(0.2)	0	(0.0)	0	(0.0)	1	(0.1)
Hypokalaemia	0	(0.0)	1	(0.1)	2	(0.5)	3	(0.2)
Hyponatraemia	0	(0.0)	4	(0.5)	1	(0.3)	5	(0.3)
Hypophosphataemia	0	(0.0)	0	(0.0)	1	(0.3)	1	(0.1)
Insulin resistant diabetes	1	(0.2)	0	(0.0)	0	(0.0)	1	(0.1)
**Musculoskeletal and connective tissue disorders**	**4**	**(1.0)**	**4**	**(0.5)**	**1**	**(0.3)**	**9**	**(0.6)**
Arthralgia	1	(0.2)	2	(0.3)	1	(0.3)	4	(0.3)
Groin pain	0	(0.0)	0	(0.0)	1	(0.3)	1	(0.1)
Muscular weakness	2	(0.5)	0	(0.0)	0	(0.0)	2	(0.1)
Musculoskeletal pain	0	(0.0)	1	(0.1)	1	(0.3)	2	(0.1)
Myalgia	2	(0.5)	0	(0.0)	0	(0.0)	2	(0.1)
Rhabdomyolysis	0	(0.0)	1	(0.1)	0	(0.0)	1	(0.1)
**Nervous system disorders**	**2**	**(0.5)**	**3**	**(0.4)**	**0**	**(0.0)**	**5**	**(0.3)**
Brain oedema	0	(0.0)	1	(0.1)	0	(0.0)	1	(0.1)
Depressed level of consciousness	0	(0.0)	1	(0.1)	0	(0.0)	1	(0.1)
Encephalopathy	1	(0.2)	0	(0.0)	0	(0.0)	1	(0.1)
Epilepsy	0	(0.0)	1	(0.1)	0	(0.0)	1	(0.1)
Headache	1	(0.2)	1	(0.1)	0	(0.0)	2	(0.1)
Meningitis noninfective	0	(0.0)	1	(0.1)	0	(0.0)	1	(0.1)
Myasthenic syndrome	0	(0.0)	1	(0.1)	0	(0.0)	1	(0.1)
**Psychiatric disorders**	**1**	**(0.2)**	**0**	**(0.0)**	**0**	**(0.0)**	**1**	**(0.1)**
Confusional state	1	(0.2)	0	(0.0)	0	(0.0)	1	(0.1)
**Renal and urinary disorders**	**0**	**(0.0)**	**1**	**(0.1)**	**2**	**(0.5)**	**3**	**(0.2)**
Dysuria	0	(0.0)	1	(0.1)	0	(0.0)	1	(0.1)
Renal failure	0	(0.0)	0	(0.0)	1	(0.3)	1	(0.1)
Renal failure acute	0	(0.0)	0	(0.0)	1	(0.3)	1	(0.1)

	MK-3475 2 mg/kg Q3W		MK-3475 10 mg/kg Q3W		MK-3475 10 mg/kg Q2W		Total	
	n	(%)	n	(%)	n	(%)	n	(%)
**Reproductive system and breast disorders**	**1**	**(0.2)**	**0**	**(0.0)**	**0**	**(0.0)**	**1**	**(0.1)**
Pelvic pain	1	(0.2)	0	(0.0)	0	(0.0)	1	(0.1)
**Respiratory, thoracic and mediastinal disorders**	**6**	**(1.5)**	**12**	**(1.5)**	**7**	**(1.8)**	**25**	**(1.6)**
Cough	0	(0.0)	0	(0.0)	1	(0.3)	1	(0.1)
Dysphonia	1	(0.2)	0	(0.0)	0	(0.0)	1	(0.1)
Dyspnoea	3	(0.7)	3	(0.4)	0	(0.0)	6	(0.4)
Hypoxia	0	(0.0)	1	(0.1)	0	(0.0)	1	(0.1)
Interstitial lung disease	0	(0.0)	2	(0.3)	0	(0.0)	2	(0.1)
Laryngeal inflammation	1	(0.2)	0	(0.0)	0	(0.0)	1	(0.1)
Pleural effusion	0	(0.0)	1	(0.1)	0	(0.0)	1	(0.1)
Pleuritic pain	0	(0.0)	0	(0.0)	1	(0.3)	1	(0.1)
Pneumonitis	2	(0.5)	6	(0.8)	5	(1.3)	13	(0.8)
**Skin and subcutaneous tissue disorders**	**2**	**(0.5)**	**6**	**(0.8)**	**6**	**(1.6)**	**14**	**(0.9)**
Erythema multiforme	0	(0.0)	1	(0.1)	0	(0.0)	1	(0.1)
Pruritus	0	(0.0)	1	(0.1)	1	(0.3)	2	(0.1)
Rash	0	(0.0)	1	(0.1)	2	(0.5)	3	(0.2)
Rash erythematous	0	(0.0)	0	(0.0)	1	(0.3)	1	(0.1)
Rash generalised	0	(0.0)	1	(0.1)	0	(0.0)	1	(0.1)
Rash maculo-papular	1	(0.2)	2	(0.3)	1	(0.3)	4	(0.3)
Rash pruritic	0	(0.0)	0	(0.0)	1	(0.3)	1	(0.1)
Stevens-Johnson syndrome	1	(0.2)	0	(0.0)	0	(0.0)	1	(0.1)
**Vascular disorders**	**1**	**(0.2)**	**3**	**(0.4)**	**0**	**(0.0)**	**4**	**(0.3)**
Hypertension	0	(0.0)	2	(0.3)	0	(0.0)	2	(0.1)
Peripheral ischaemia	1	(0.2)	0	(0.0)	0	(0.0)	1	(0.1)
Vasculitis	0	(0.0)	1	(0.1)	0	(0.0)	1	(0.1)

Every subject is counted a single time for each applicable row and column.
A system organ class or specific adverse event appears on this report only if its incidence in one or more of the columns meets the incidence criterion in the report title, after rounding.
Include all treated subjects in P001 Parts B1, B2, B3, D, C, F1, F2, and F3 and all subjects in P002 treated with pembrolizumab in the original phase.
(MK-3475 P001 Database Cutoff Date for Melanoma: 18APR2014).
(MK-3475 P001 Database Cutoff Date for Lung: 29AUG2014).
(MK-3475 P002 Database Cutoff Date: 12MAY2014).

Refer to the current version of the pembrolizumab Investigator's Brochure for additional details.

1.4.4 Overview of bavituximab

Bavituximab is a genetically engineered immunoglobulin gamma 1 (IgG1) chimeric (human/mouse) mAb containing the variable region sequences of the murine PStargeting mouse antibody 3G4(2aG4) and human IgG1 k constant region sequences that targets PS after binding to 2-glycoprotein 1 (2GP1). PS is a highly immunosuppressive molecule typically expressed on the inner leaflet of the plasma membrane of normal cells but "flips" and becomes externalized to the outer leaflet of the plasma membrane on cells that line tumor blood vessels, tumor cells and exosomes in the tumor microenvironment creating a specific target for anticancer treatments.

Bavituximab is a novel immunotherapeutic approach because its primary mechanism of action involves modulating the tumor microenvironment from a primarily immunosuppressive, angiogenesis-promoting state (with infiltrating myeloid-derived

suppressor cells [MDSCs] and M2-macrophages) to an immune activating state (with long-term tumor specific-immunity facilitated by M1 macrophages, mature dendritic cells, and activated T lymphocytes) that drives both innate and adaptive immunity. Bavituximab blocks the immunosuppressive signaling of myeloid derived cells and sends and alternate immune activation signal leading to an adaptive T-cell mediated anti-tumor response. In addition, through activation of innate immunity (i.e., M1 macrophages and natural killer [NK] cells), bavituximab causes tumor cell destruction and the selective shutdown of pre-existing tumor blood vessels expressing PS via antibody-dependent cell mediated cytotoxicity.

1.4.5 Bavituximab non-clinical experience

Nonclinical experiments have shown that antibody-mediated PS targeting can override PS-mediated immunosuppression, reactivate innate tumor immunity, and evoke adaptive antitumor immunity. In genetically modified nonclinical murine tumor models, PS targeting induces potent tumor-specific T-cell immunity in the transgenic adenocarcinoma mouse prostate tumor model such that the combination of anti-PS with castration cured 35% of the animals, compared to none in control groups. In separate pre-clinical studies, the use of antibodies against PD-1 or PD-L1 have demonstrated similar activity as single agents and when combined with other immune oncology agents.

To support clinical development, bavituximab was evaluated in a series of dose range finding, repeated dose and specialized IV toxicology studies in appropriate species and in vitro systems. In vivo toxicology studies consisted of nonGLP dose range finding studies in the Sprague-Dawley® (SD) rat, rabbit and cynomolgus monkey as single IV dose administration, followed by GLP 8-week once-weekly dosing toxicology studies in the rat and monkey. The reversibility of any observed toxicity was determined in recovery studies included as part of the protocol for selected studies. The in vivo studies employed intravenous bolus dosing of bavituximab according to the current clinical dosing regimen and route of administration. Toxicokinetic (TK) analyses were conducted in rats and monkeys to facilitate correlating observed toxicity to systemic exposure to bavituximab.

The nonclinical safety assessment program that has been undertaken fully supports the continual use of bavituximab in clinical trials in oncology patients. The nonclinical effects of bavituximab have been assessed in a comprehensive panel of studies, addressing the relationships of pharmacology, pharmacokinetics/toxicokinetics, immunogenicity and toxicology; the target tissues for toxicity; the NOAELs and LOAELs; the noninvasive monitors for the key toxicologic responses; and the reversibility of adverse effects. Among the recovery animals at the high dose (100 mg/kg) in the rat toxicology study, there remained evidence of microscopic sub endocardial inflammation and/or fibrosis, however, there were no coagulation abnormalities. Similarly, bavituximab-related microscopic changes in monkeys were confined to a mild, thrombus within the right ventricle of one male at 100 mg/kg and no coagulation abnormalities in the recovery phase. Results from the bavituximab nonclinical program to date indicate a therapeutic benefit of combining bavituximab with conventional (chemotherapy) or immunotherapy (checkpoint blockade) for continued clinical evaluations and support the safety of the proposed dose of 3 mg/kg bavituximab administered intravenously once weekly in patients.

Detailed information about the comprehensive nonclinical safety, immunology, and pharmacokinetic (PK) testing program is provided in the current version of the bavituximab Investigator's Brochure.

1.4.6 Bavituximab clinical experience

To date, the clinical development program of bavituximab has enrolled approximately 900 patients, of whom over 600 patients have been treated with bavituximab. Most patients were treated with combination therapy in oncology studies.

In the oncology program, where bavituximab was given in combination with cytotoxic chemotherapy in all but 26 patients, common adverse events (20%) were reported in patients receiving bavituximab include the following: nausea, alopecia, fatigue, diarrhea, neutropenia, anemia, pyrexia, and asthenia. Many of the reported common AEs were considered to be related to the concomitant administration of chemotherapy or the patient's underlying malignancy.

AEs reported with bavituximab clinical trials and a summary of clinical experience are described in the current version of the bavituximab Investigator's Brochure.

Phase I and Ib single-agent studies of bavituximab in combination with chemotherapy have been completed in patients with advanced cancer. Based on efficacy results from these studies and on PK data (which determined the maximum binding of β2-GP1), a dose of 3 mg/kg bavituximab was determined and selected for further clinical study. The studies also showed the tolerability profile to be consistent with expectations based on animal studies, and demonstrated adequate efficacy to warrant further study. Several Phase II studies of bavituximab in combination with chemotherapy have been completed. A Phase III study to evaluate the efficacy and safety of bavituximab combined with docetaxel in patients with previously treated Stage IIIb/IV non-squamous NSCLC is ongoing.

In a Phase II, randomized, double-blind study, 121 patients with previously treated, locally advanced, or metastatic non-squamous NSCLC were randomized to receive docetaxel plus placebo, docetaxel plus 1 mg/kg bavituximab, or docetaxel plus 3 mg/kg bavituximab. Due to a labeling error between the placebo and 1 mg product, efficacy analyses were performed comparing the 3 mg/kg group to a pooled control group of 1 mg/kg and placebo. There was an improvement of almost 50% in ORR favoring the 3 mg/kg bavituximab group compared to the pooled (placebo and 1 mg/kg) group, although this trend was not statistically significant. There was a positive trend in median PFS favoring the 3 mg/kg bavituximab group compared to the pooled control group (placebo and 1 mg/kg). Consistent with the trends observed in ORR and PFS, there was an improvement (60%) in median OS and hazard ratio (0.66) favoring the 3 mg/kg bavituximab group compared to the pooled control group (placebo and 1 mg/kg). Though the differences were not statistically significant, there was clear and persistent separation in the Kaplan-Meier survival curves.

Overall, results from Phase I and Phase II studies suggested a beneficial treatment effect of bavituximab, and results were consistent among studies. The overall safety profile of

bavituximab was acceptable, and the safety data were consistent with those observed in other clinical studies. Combination therapy did not substantially increase the risks of side effects. The adverse events observed in the phase 1 study of bavituxumab and sorafenib in patients with hepatocellular cancer are summarized in the table below:

Adverse events observed in the Phase 1 study of bavituxumab plus sorafenib attributed to be Possibly, Probably, and Definitely Related to treatment n=9:

Event Term	Grade 1	Grade 2	Grade 3	Grade 4
Hand/Foot Syndrome	3	2	0	0
Hypertension	1	0	2	0
Pruritus	3	0	0	0
Fatigue	2	0	0	0
Headache	2	0	0	0
Hyponatremia	1	0	1	0
Diarrhea	1	0	0	0
Erythema R index finger	1	0	0	0
Generalized muscle weakness	1	0	0	0
Leukopenia	0	1	0	0
Mucositis	1	0	0	0
Neutropenia	0	1	0	0
Pain; Bilateral Feet	1	0	0	0
Peripheral Neuropathy	1	0	0	0
Right foot tenderness	1	0	0	0
Thrombocytopenia	1	0	0	0
Hypocalcemia	0	1	0	0
Nausea	1	0	0	0
Vomiting	0	1	0	0
Anorexia	1	0	0	0

Thus the potential additional benefits of combination therapy are likely to outweigh the risk of bavituximab-related adverse events. Refer to the current version of the bavituximab Investigator's Brochure for additional details.

1.5 STUDY RATIONALE

The major challenge to effective immunotherapy is to overcome multiple pathways that inhibit innate or adaptive immune activation. Although anti-tumor effects have been observed in a percentage of cancer patients by blockade of immune checkpoints like PD-1; only a limited number of patients benefit from this therapy. Recent preclinical results indicate that the combination of PS targeting with PD-1 blockade demonstrated additive to synergistic antitumor efficacy in mice bearing K1735 and B-16F10 melanoma as well as EMT-6 and E077.1 breast carcinoma. This antitumor efficacy was associated with increases in intratumoral activated CD8 T cells (Ki67, IL-2 and Granzyme B positive), a reduction of M2 macrophages and MDSCs, and increased tumor reactive T cells in the spleen. Preclinical results indicate that antibody-mediated PS targeting with bavituximab reverses PS-mediated immunosuppression and initiates therapeutically effective adaptive antitumor immunity. (31,32,33) Thus, treatment with bavituximab in combination with a downstream immune checkpoint antagonist like pembrolizumab, may result in robust and long- lasting antitumor immunity that significantly improves clinical outcomes of duration and levels of response.

Novel combinations are being explored for synergistic mechanisms of action to increase the limited anti-tumor immunity in cancer patients and evaluate the potential for new therapies for patients who are refractory to or progress on checkpoint blockade.

Clinical studies with bavituximab in combination with chemotherapy in HCC, NSCLC, breast, and other cancers have shown signs of improved antitumor activity relative to chemotherapy alone.

Based on these data, and the preclinical results demonstrating synergism between bavituximab and anti-PD-1/PD-L1 therapy noted above, this study will test the hypothesis that bavituximab can improve the outcome for patients with HCC treated with pembrolizumab, a PD-1 inhibitor.

Since the efficacy of sorafenib, the only currently FDA approved systemic therapy, for advanced HCC is modest at best with overall survival and radiologic time to progression increased by under 3 months compared to placebo the need for more efficacious therapies is paramount. In addition, the adverse effects of sorafenib are high enough that over one-third of patients discontinue therapy due to drug-related toxicity.

1.6 RATIONALE FOR TREATMENT BEYOND PROGRESSION

For cytotoxic agents, an increase in tumor burden of the appearance of new lesions signals progressive disease and has become synonymous with drug failure. An assessment of progressive disease (PD) signifies the need for alternative management and discontinuation of current therapy. Recent experience indicates that the parameters for cytotoxic drug response assessment may not be sufficient to fully characterize the activity of targeted therapies, biologics, and/or immunotherapy agents.

Immunotherapeutic agents such as pembrolizumab and bavituximab, produce antitumor effects by inducing cancer-specific immune responses or by modifying native immune processes. Traditional parameters for defining PD may not adequately assess the activity of immunotherapeutic agents because a) the appearance of measurable antitumor activity may take longer for immune therapies than for cytotoxic therapies; b) responses to immune therapies may occur after conventional PD; c) discontinuation of immune therapy may not be appropriate in some cases, unless PD is confirmed (as is usually done for response); d) allowance for clinically insignificant PD (i.e. small new lesions in the presence of other responsive lesions) is recommended; and e) durable stable disease (SD) may represent antitumor activity. (34)

In a phase I clinical trial (KEYNOTE-001) administering pembrolizumab in advanced melanoma, 14% of patients (84/600) experienced progressive disease per RECIST 1.1 but non-progressive disease per immune-related response criteria (irRC). The 84 patients with progressive disease by RECIST but non-progressive disease by irRC had a longer overall survival than the 177 patients with progressive disease per both criteria, which suggests that RECIST might underestimate the benefit of pembrolizumab in approximately 15% of patients. (35) These data suggest that patients may benefit from receiving treatment beyond initial evidence of radiographic progression and thus support the use of modified response criteria on the basis of immune-related response patterns. The data support study treatment with pembrolizumab and bavituximab beyond progression in this study under the parameters outlined in section 7.2.

1.7 RATIONALE FOR EFFICACY ENDPOINTS

Overall response rate (ORR) is the primary endpoint for this study (see section 3.2). ORR will be estimated as the number of responders (either partial or complete response) as a percent of the number of eligible participants who received at least one dose of bavituximab and pembrolizumab. Best overall response requires confirmation based on RECIST 1.1 on one imaging test greater than or equal to 9 weeks. ORR is generally favored as a progression endpoint in solid tumor treatment and has been associated with overall survival in HCC. (14)

1.8 RESEARCH HYPOTHESIS

It is our hypothesis that combination therapy pembrolizumab and bavituximab in patients with locally advanced HCC not amenable to curative therapies will provide greater efficacy than historical systemic therapy alone with a tolerable safety profile.

2.0 STUDY OBJECTIVES

2.1 PRIMARY OBJECTIVE

2.1.1 To determine the overall response rate (ORR) of combination pembrolizumab and bavituximab in patients with advanced HCC.

2.2 SECONDARY OBJECTIVES

2.2.1 To determine overall survival, 6-month progression free survival, and duration of response of combination pembrolizumab and bavituximab compared to historical controls;

2.2.2 To determine the safety and tolerability of combination pembrolizumab and bavituximab as measured by rates of adverse events according to the CTCAE v. 4.03;

2.3 EXPLORATORY OBJECTIVES

2.3.1 To determine treatment response with pre- and intra-treatment biopsies and plasma/serum. Correlative studies will be performed on the collected tissue and blood samples to determine if predictive markers of response can be generated

3.0 STUDY DESIGN

3.1 DESIGN

This is a non-randomized, open-label, multi-site phase II therapeutic trial of pembrolizumab and bavituximab in patients with locally advanced HCC. Locally advanced or metastatic HCC is defined as disease that is not amenable to surgical and/or locoregional therapies. Subjects must not have received prior systemic therapy for advanced HCC in keeping with the first-line setting of this study.

Overall response rate (ORR) as assessed by the investigators and using RECIST 1.1 criteria, will be the primary endpoint.

<u>Screening Phase</u>

Prior to initiation of treatment, all patients will sign an informed consent form. Only patients that meet all inclusion criteria and none of the exclusion criteria will be eligible for treatment.

<u>Treatment Phase</u>

The treatment phase for each patient will begin on the initiation of treatment with both pembrolizumab and bavituximab study drug on Cycle 1 Day 1 (C1D1). Patients will continue study treatment until disease progression (or until discontinuation of study therapy in patient receiving pembrolizumab and bavituximab beyond progression), discontinuation due to toxicity, withdrawal of consent, or the study ends. Patients with radiological progression using RECIST may continue on study treatment if in the investigator’s assessment the patient is experiencing clinical benefit and tolerating the treatment. A safety follow-up is mandatory at 30 days post the last dose of Bavituximab.

Efficacy, safety, and correlative assessments will be performed as outlined in the Schedule of Visits and Procedures (section 6.0**)**.

Dose Limiting Toxicity:

Initially, enrollment will be limited to 10 evaluable patients with no more than two patients enrolled per week. A safety committee comprised of the investigators and institutional GI Disease Orientated Team will monitor the occurrence of dose limiting toxicities (DLTs) for the first 10 patients prior to enrolling the remainder of the trial following the DSMC guidelines. The period for evaluating DLTs will be from the time of the first administration of study treatment through Study Day 28. DLTs will follow the guidelines provided in the Common Terminology Criteria for Adverse Events (CTCAE) version 4.03.

If three or more DLTs occur during this period, enrollment will be suspended and the DSMC will make a determination of whether to reduce the bavituximab dose to 1 mg/kg. After the DLT safety assessment period for the initial 10 patients, enrollment may proceed for the remainder of the trial. Patient safety is evaluated on a regular basis by the institutional data safety monitoring committee (DSMC). A DLT will be defined as any treatment related toxicity in the list below that occurs during the DLT evaluation period. Toxicity that is clearly and directly related to the primary disease or to another etiology is excluded from this definition. The following will be considered DLTs:

- Any grade 4 immune-related adverse events (irAE)
- Any ≥ grade 3 colitis
- Any grade 3 or 4 noninfectious pneumonitis irrespective of duration
- Any grade 2 pneumonitis that does not resolve to grade 1 within 5 days of the initiation of maximum supportive care
- Any grade 3 irAE, excluding colitis or pneumonitis, that does not downgrade to grade 2 within 5 days of the event despite optimal medical management including systemic corticosteroids or does not downgrade to grade 1 or baseline within 14 days
- Liver transaminase elevation >8 x ULN or total bilirubin > 5 x ULN
- Any ≥ grade 3 non-irAE, except for the exclusions listed below The definition excludes the following conditions:
    - Grade 3 fatigue ≤ 7days
    - Grade 3 endocrine disorder (thyroid, pituitary, and/or adrenal insufficiency) that is managed with or without systemic corticosteroid therapy and/or hormone replacement therapy and the patient is asymptomatic
    - Grade 3 inflammatory reaction attributed to a local antitumor response o Concurrent vitiligo or alopecia of any grade
    - Grade 3 infusion-related reaction (first occurrence and in the absence of steroid prophylaxis) that resolves within 6 hours with appropriate clinical management
    - Grade 3 or 4 neutropenia that is not associated with fever or systemic infection that improves by at least 1 grade within 3 days. Grade 3 or 4 febrile neutropenia will be considered a DLT regardless of duration or reversibility
    - Grade 3 or 4 lymphopenia
    - Grade 3 thrombocytopenia that is not associated with clinically significant bleeding that requires medical intervention, and improves by at least 1 grade within 3 days

- Isolated grade 3 electrolyte abnormalities that are not associated with clinical signs or symptoms and are reversed with appropriate maximal medical intervention within 3 days
- Isolated grade 3 amylase or lipase abnormalities that are not associated with clinical signs/symptoms or findings on imaging consistent with pancreatitis.

Survival follow-up phase
Patients who are no longer receiving study treatment on this clinical trial, for any reason, will enter survival follow-up, regardless of whether they have initiated subsequent anticancer therapy. Survival follow-up information (by chart review, phone call or clinic visits) will be collected approximately every 12 weeks until death, loss to follow-up, withdrawal of consent or study termination.

3.2 ENDPOINTS AND SAMPLE SIZE JUSTIFICATION

The primary endpoint of the phase II trial will be overall response rate (ORR) of HCC patients receiving at least one dose of pembrolizumab and bavituximab. ORR is defined as number of responders (either complete or partial response as best overall response) of eligible patients. Best overall response requires confirmation imaging greater than or equal to 9 weeks after initial best response. The historical ORR for systemic therapy with the PD-1 inhibitor, nivolumab, in the phase I/II CHECKMATE trial is between 15 and 20% (42 of 262 patients currently examined)[1].

We used minimax two-stage design method to develop phase II trial to compare the ORR of 0.35 (alternative hypothesis) for the treatment cohort versus 0.15 (null hypothesis) for the historical control. In the first stage 15 patients will be accrued. If there are 3 or more responses then 13 additional patients will be accrued for a total of 28 patients. The null hypothesis will be rejected if 8 or more responses are observed in 28 patients. This design yields a type I error rate of 0.05 and power of 0.80 when the true response rate is 0.35%.

PFS will be estimated using the Kaplan-Meier method, and Greenwood's formula will be used to calculate the standard error of the corresponding Kaplan-Meier estimate and 95% confidence interval. Secondary endpoints of efficacy are to evaluate OS. The statistical methods used for the analysis of PFS will be used for the analysis of OS.

For exploratory endpoints including pathology- and laboratory-correlative studies, Cox regression analysis will be conducted to investigate the association between ORR and parameters from correlative studies.

4.0 SUBJECT ELIGIBILITY

Eligibility waivers are not permitted. Subjects must meet all of the inclusion and exclusion criteria to be registered to the study. Study treatment may not begin until a subject is registered. (See Section 13.3)

4.1 INCLUSION CRITERIA

4.1.1 Patient must have a histologically confirmed diagnosis hepatocellular carcinoma; known fibrolamellar HCC, sarcomatoid HCC or mixed cholangiocarcinoma and HCC will be excluded

4.1.2 Locally advanced or metastatic disease

4.1.2.1 Patients with locally advanced or metastatic disease not eligible for surgical and/or locoregional therapies.

4.1.2.2 Measurable disease, as defined as lesions that can accurately be measured in at least one dimension according to RECIST version 1.1.

4.1.3 Child-Pugh Score A

4.1.4 Age ≥ 18 years;

4.1.5 ECOG Performance score of 0-1;

4.1.6 Life expectancy greater than 6 months;

4.1.7 Following baseline laboratory values:

4.1.7.1 Total bilirubin ≤ 2.0 mg/ml

4.1.7.2 INR ≤ 1.7;

4.1.7.3 Hgb ≥ 8.5 g/dl;

4.1.7.4 AST, ALT ≤5 times ULN;

4.1.7.5 Platelet count ≥ 50,000/mm^3;

4.1.7.6 Serum creatinine ≤ 1.5 mg/dL or creatinine clearance ≥ 50 mL/min;

4.1.7.7 Albumin ≥ 2.5 g/dl;

4.1.7.8 Absolute neutrophil ≥ 1,500 cells/mm$^{3;}$

4.1.8 Male and female subjects of child bearing potential must agree to use an adequate method of contraception beginning Cycle 1 Day 1 through the course of the study, and 120 days after the last dose of study medication;

4.1.9 Women of childbearing potential must have a negative pregnancy test;

4.1.10 Subjects are eligible to enroll if they have non-viral-HCC, or if they have HBVHCC, or HCV-HCC defined as follows:

HBV-HCC: Controlled (treated) hepatitis B subjects will be allowed if they meet the following criteria: Antiviral therapy for HBV must be given for at least 12 weeks and HBV viral load must be less than 100 IU/mL prior to first dose of study drug. Subjects on active HBV therapy with viral loads under 100 IU/ml should stay on the same therapy throughout study treatment. Subjects who are anti-HBc (+), negative for HBsAg, negative for anti-

HBs, and have an HBV viral load under 100 IU/mL do not require HBV anti-viral prophylaxis.

HCV-HCC: active or resolved HCV infection as evidenced by detectable HCV RNA or antibody. Patients who have failed HCV therapy as evidenced by detectable HCV RNA will be eligible. Subjects with chronic infection by HCV who are treated (successfully or treatment failure) or untreated are allowed on study. In addition, subjects with successful HCV treatment are allowed as long as there are ≥4 weeks between completion of HCV therapy and start of study drug. Successful HCV treatment definition: SVR_{12}.

4.1.11 Prior therapy is allowed provided the following are met: at least 4 weeks since prior locoregional therapy including surgical resection, chemoembolization, radiotherapy, or ablation. Provided target lesion has increased in size by 25% or more or the target lesion was not treated with locoregional therapy. Patients treated with palliative radiotherapy for symptoms will be eligible 1 week after treatment as long as the target lesion is not the treated lesion.

4.2 EXCLUSION CRITERIA

4.2.1 Prior liver transplant;

4.2.2 Patient who has received previous systemic therapy for HCC;

4.2.3 Clinically significant, uncontrolled heart disease and/or recent events including any of the following within 12 months of screening date:

4.2.3.1 History of acute coronary syndromes (including myocardial infarction, unstable angina, coronary artery bypass grafting,

4.2.3.2 Coronary angioplasty, or stenting) or symptomatic pericarditis;

4.2.3.3 History of documented congestive heart failure (New York Heart Association functional classification III-IV);

4.2.3.4 Documented cardiomyopathy;

4.2.3.5 Left ventricular ejection fraction <40% as determined by MUGA scan or ECHO (MUGA and ECHO are not required for enrollment);

4.2.4 Known human immunodeficiency virus (HIV) positive (testing not required);

4.2.5 History of thromboembolic events (including both pulmonary embolism and deep venous thrombus but not including tumor thrombus) within the last 6 months;

4.2.6 Hypersensitivity to IV contrast; not suitable for pre-medication;

4.2.7 Active or fungal infections requiring systemic treatment within 7 days prior to screening;

4.2.8 Known history of, or any evidence of, interstitial lung disease or active noninfectious pneumonitis;

4.2.9 Evidence of poorly controlled hypertension which is defined as systolic blood pressure >150 mmHg or diastolic pressure >90 mmHg despite optimal medical management;

4.2.10 Pre-existing thyroid abnormality with thyroid function that cannot be maintained in the normal range with medication;

4.2.11 Active, known, or suspected autoimmune disease with the following exceptions:

i) Subjects with vitiligo, type I diabetes mellitus, resolved childhood asthma or atopy are permitted to enroll;
ii) Subjects with suspected autoimmune thyroid disorders may be enrolled if they are currently euthyroid or with residual hypothyroidism requiring only hormone replacement.
iii) Subjects with psoriasis requiring systemic therapy must be excluded from enrollment.

4.2.12 Patient has any other concurrent severe and/or uncontrolled medical condition that would, in the investigator's judgment, cause unacceptable safety risks, contraindicate patient

participation in the study or compromise compliance with the protocol (e.g. chronic pancreatitis, active untreated or uncontrolled fungal, bacterial, or viral infections, etc.);

4.2.13 Known history of active bacillus tuberculosis;

4.2.14 Subjects with a condition requiring systemic treatment with either corticosteroids (> 10 mg/day prednisone equivalent) or other immunosuppressive medications within 14 days of study administration. Inhaled or topical steroids and adrenal replacement doses >10 mg/day prednisone equivalents are permitted in the absence of autoimmune disease;

4.2.15 Patient who has received radiotherapy ≤ 4 weeks prior to study entry. Palliative radiotherapy for symptomatic control is acceptable (if completed at least 2 weeks prior to study drug administration and no additional radiotherapy for the same lesion is planned);

4.2.16 Patient has had major surgery within 14 days prior to starting study drug or has not recovered from major side effects (tumor biopsy is not considered as major surgery);

4.2.17 Clinically apparent ascites on physical examination, ascites present on imaging studies is allowed;

4.2.18 Patient has a known hypersensitivity to any of the excipients of bavituximab or pembrolizumab or monoclonal antibody;

4.2.19 Active gastrointestinal bleeding within previous 2 months;

4.2.20 History of any condition requiring anti-platelet therapy (aspirin >300 mg/day, clopidogrel >75 mg/day);

4.2.21 Prisoners or subjects who are involuntarily incarcerated;

4.2.22 Symptomatic or clinically active brain metastases;

4.2.23 Pregnant or nursing (lactating) women, where pregnancy is defined as the state of a female after contraception and until the termination of gestation, confirmed by a positive hCG laboratory test;

4.2.24 Prior immunotherapy including anti-PD-1, anti-PD-L1, or anti-PD-L2 agents;

4.2.25 Has dual active HBV infection (HBsAg (+) and /or detectable HBV DNA) and HCV infection (anti-HCV Ab(+) and detectable HCV RNA) at study entry.

5.0 TREATMENT PLAN

5.1 OVERVIEW

STUDY TREATMENT

For this study, the term “study treatment” refers to the combination of pembrolizumab and bavituximab.

Bavituximab will be provided by OncXerna Therapeutics (Boston, MA) as a 5ml

vial of sterile solution in borosilicate type I glass vials that contains 120 mg bavituximab (24 mg/ml), 10mM acetate at pH 5.0, and water for injection, United States Pharmacopeia.

Pembrolizumab will be provided by Merck Pharmaceutical, Inc (Kenilworth, NJ) as a white to off while lyophilized powder (50 mg/vial) or as a liquid solution (100 mg/vial) in Type I glass vials intended for single use only.

5.2 TREATMENT REGIMEN SCHEDULE AND DOSE ADJUSTMENTS

Pembrolizumab and bavituximab will identified as investigational product. Patients will receive 200 mg pembrolizumab every three weeks in combination with 3 mg/kg of bavituximab weekly. Each patient will be dosed at a frequency until second confirmed disease progression, discontinuation due to toxicity, withdrawal of consent, or the study ends.

Table 5.1 Dose and Treatment Schedule

Study Treatment	Pharmaceutical form and route of administration	Total Dose	Frequency and/or Regimen
Pembrolizumab	Intravenous	200 mg	Once every three weeks
Bavituximab	Intravenous	3 mg/kg	Once every week

Pembrolizumab will be administered as a flat-fixed dose, and not by body weight or body surface area. Bavituximab will be administered at 3 mg/kg body weight and diluted with normal saline to a volume no less than 100 ml. The total dose is only required to be recalculated if there is a ≥ 10% change in weight from C1D1. There will be otherwise no modification of dose level or schedule or bavituximab or pembrolizumab treatment.

Pre-medication(s) is not required, but can be added after C1 in the event of an infusion reaction. Follow packet insert or institutional guidelines for pre-medication regimen.

5.3 THERAPEUTIC/DIAGNOSTIC AGENTS

5.3.1 Bavituximab

<u>IND Number: 135123</u>

<u>Finished Product</u>

Presentation and Composition

Bavituximab is supplied as a sterile, preservative-free solution with 10 mM acetate at pH 5.0 and diluted with 0.9% (w/v) saline (normal saline) to a final volume of 100 mL.

Storage

Bavituximab is stored at 2°C to 8°C. Once diluted, it should be stored at room temperature and used within 8 hours.

Availability and Accountability

OncXerna Therapeutics in Boston, MA will provide bavituximab. The pharmacist or designee must keep an accurate accounting of the number of investigational units received from OncXerna Therapeutics, dispensed to patients, and returned to OncXerna Therapeutics during and at the completion of the study. A detailed inventory must be completed for the study medication. The study medication must be dispensed only by an appropriately qualified person to patients in the study. The medication is to be used in accordance with the protocol in patients who are under the direct supervision of an investigator. All unused clinical study medications/treatments will be returned to OncXerna Therapeutics.

Administration

Bavituximab will be administered weekly.

Infusion preparation and administration are to be performed as follows:

1. Draw up the volume of a vial using a sterile syringe attached to an appropriate needle (a vented spike or other appropriate transfer device may be used). Fill treatment into a sterile glass bottle or plastic IV bag.
2. Using aseptic techniques, repeat procedure until the calculated volume has been put in to the container. Bring the final volume to 100 mL using 0.9% Sodium Chloride Injection, USP.
3. Administer through a low protein binding 0.2-micrometer in-line filter (placed as proximal to the patient as practical).
4. Affix the infusion line and prime it with infusate before starting the infusion. Infuse the solution intravenously over 90 (± 10) minutes. No reduction in infusion time will be permitted. Flush the line with normal saline after infusion.

5.3.2 PEMBROLIZUMAB

IND Number 135123

Finished Product

Presentation and Composition

Pembrolizumab is supplied as a lyophilized powder and reconstituted with 2.3 ml sterile water for injection to yield a 2.4 ml solution containing 25 mg/ml of pembrolizumab at pH 5.2-5.8.

Storage

Pembrolizumab powder for solution for infusion vials should be stored at 2°C to 8°C.

Prior to reconstitution, the vial of lyophilized powder can out of refrigeration (temperatures at or below 25°C for up to 24 hours. Following reconstitution with sterile water for injection, pembrolizumab infusion solutions should be prepared in 0.9% sodium chloride

injection or regional equivalent and the final concentration of pembrolizumab in the infusion solutions should be between 1mg/ml and 10 mg/ml.

Availability and Accountability

Merck Pharmaceuticals will provide pembrolizumab. The pharmacist or designee must keep an accurate accounting of the number of investigational units received from Merck dispensed to patients, and returned to Merck during and at the completion of the study. A detailed inventory must be completed for the study medication. The study medication must be dispensed only by an appropriately qualified person to patients in the study. The medication is to be used in accordance with the protocol in patients who are under the direct supervision of an investigator. All unused clinical study medications/treatments will be returned to Merck.

Administration

Pembrolizumab will be administered every three weeks. Pembrolizumab should be administered over 30 minutes, with a window of -5 and +10 minutes, using an infusion pump. Pembrolizumab should be administered at least 30 minutes after the bavituximab infusion (on days when both are administered). The infusion line should be flushed with 50 ml normal saline prior to pembrolizumab infusion.

Please refer to packet insert or local institutional guidelines.

5.4 MONITORING OF STUDY TREATMENT DOSE ADMINISTRATION

In the event of a ≤ grade 2 infusion-related reaction, the infusion rate of study drug may be decreased by 50% or interrupted until resolution of the event (up to 4 hours) and re-initiated at 50% of the initial rate until completion of the infusion. For patients with a ≤ grade 2 infusion-related reaction, subsequent infusions may be administered at 50% of the initial rate. Acetaminophen and/or antihistamines, corticosteroids or equivalent medication may be administered at the discretion of the principal investigator. If the infusion-related reaction is ≥ grade 3, study drug will be discontinued.

5.5 TREATMENT DURATION

Study treatment with pembrolizumab and bavituximab will continue until protocol specified 2nd occurrence disease progression, unacceptable toxicity, maximum treatments (35) reached for pembrolizumab, death or discontinuation from the study treatment for any other reason.

Bavituximab may continue past pembrolizumab administration. All participants who stop study treatment with SD or better may be eligible for up to an additional 17 cycles (approximately 1 year) of pembrolizumab treatment if they progress after stopping study treatment from the initial treatment phase. This retreatment is termed the Second Course Phase of this study and is only available if the study remains open and the participant meets the following conditions:

Either

- Stopped initial treatment with study treatment after attaining an investigator-determined confirmed CR based on RECIST 1.1, and
- Was treated with at least 8 cycles of study treatment before discontinuing treatment, and

- Received at least 2 treatments with pembrolizumab beyond the date when the initial CR was declared

OR

- Had SD, PR, or CR and stopped study treatment after completion of 35 administrations (approximately 2 years) of study treatment for reasons other than disease progression or intolerability

AND

- Experienced an investigator-determined radiographic disease progression by RECIST 1.1 after stopping initial treatment, and
- No new anticancer treatment was administered after the last dose of study treatment, and
- The participant meets all of the safety parameters listed in the inclusion criteria and none of the safety parameters listed in the exclusion criteria, and
- The study is ongoing

An objective response or disease progression that occurs during the Second Course Phase for a participant will not be counted as an event for the primary analysis of either endpoint in this study. **Note: patients must have measurable disease at the start of protocol treatment to be eligible for this provision.*

5.6 CONCOMITANT MEDICATIONS-ALLOWED AND PROHIBITED

Medications or vaccinations prohibited in the exclusion criteria are not allowed. If there is a clinical indication for one of these or other medications or vaccinations specifically prohibited during the trial, discontinuation from trial therapy or vaccination may be required.

5.6.1 Acceptable concomitant medications

All treatments that the principal investigator considers necessary for a subject's welfare may be administered at the discretion of the investigator in keeping with the community standards of medical care. All concomitant medications will be recorded on the case report form (CRF) including all prescription, over-the-counter (OTC), herbal supplements, and IV medications and fluids if changes occur during the trial period, documentation of drug dosage, frequency, route, and date may also be included on the CRF.

All concomitant medications received within 28 days before the first dose of trial treatment and 30 days after the last dose of trial treatment should be recorded.

5.6.2 Prohibited concomitant medications

Subjects are prohibited from receiving the following therapies during the screening and treatment phase of this trial:

- Antineoplastic systemic chemotherapy or biological therapy;
- Immunotherapy not specified in this protocol;
- Chemotherapy not specified in this protocol;
- Investigational agents other than pembrolizumab and bavituximab;

- Radiation therapy;
- Live vaccines within 30 days prior to the first dose of trial treatment and while participating in the trial. Examples of the live vaccines included, but are not limited to, the following: measles, mumps, rubella, varicella/zoster, yellow fever, rabies, BCG, and typhoid vaccine;
- Systemic glucocorticoids for any purpose other than to modulate symptoms from an event of clinical interest of suspected immunologic etiology.

There are no prohibited therapies during the post-treatment follow-up phase.

5.7 DOSE MODIFICATION GUIDELINES

Adverse events (both non-serious and serious) associated with pembrolizumab and bavituximab exposure may represent an immunologic etiology. The adverse events may occur shortly after the first dose or several months after the last dose of treatment. Drug therapy must be withheld for drug-related toxicities and severe or life-threatening adverse events as per Table 5.2 below. See section 5.8 for supportive care guidelines, including the use of corticosteroids.

Table 5.2 Dose Modification Guidelines for Drug-Related Adverse Events

General Instructions:

1. Corticosteroid taper should be initiated upon AE improving to Grade 1 or less and continue to taper over at least 4 weeks.
2. Treatment can be resumed after AE has been reduced to Grade 1 or less and corticosteroid has been tapered.
3. Pembrolizumab should be permanently discontinued if AE does not resolve within 12 weeks of last corticosteroid dose or corticosteroids cannot be reduced to ≤10 mg prednisone or equivalent per day within 12 weeks.
4. For severe and life-threatening irAEs, IV corticosteroid should be initiated first followed by oral steroid. Other immunosuppressive treatment should be initiated if irAEs cannot be controlled by corticosteroids.

Immune-related AEs	Toxicity grade or conditions (CTCAEv4.0)	Action	Event Management	Monitor and Follow-Up
Pneumonitis	Gr 2	Withhold	Administer corticosteroids (initial dose of 1-2 mg/kg prednisone or equivalent) followed by taper	• Monitor participants for signs and symptoms of pneumonitis<br>• Evaluate participants with suspected pneumonitis with radiographic imaging and initiate corticosteroid treatment<br>• Add prophylactic antibiotics for opportunistic infections
	Gr 3 or Gr 4, or recurrent Gr 2	Permanently discontinue		
Diarrhea/ Colitis	Gr 2 or Gr 3	Withhold	Administer corticosteroids (initial dose of 1-2 mg/kg prednisone or equivalent) followed by taper	• Monitor participants for signs and symptoms of enterocolitis (ie, diarrhea, abdominal pain, blood or mucus in stool with or without fever) and of bowel perforation (ie, peritoneal signs and ileus).<br>• Participants with ≥ Grade 2 diarrhea suspecting colitis should consider GI consultation and performing endoscopy to rule out colitis.<br>• Participants with diarrhea/colitis should be advised to drink liberal quantities of clear fluids. If sufficient oral fluid intake is not feasible, fluid and electrolytes should be substituted via IV infusion.
	Gr 4	Permanently discontinue		

Immune-related AEs	Toxicity grade or conditions (CTCAEv4.0)	Action	Event Management	Monitor and Follow-Up
AST/ ALT elevation or Increased bilirubin	Gr 2	Withhold	• Administer corticosteroids (initial dose of 1-2 mg/kg prednisone or equivalent) followed by taper	• Monitor with liver function tests (weekly is recommended) until resolves to baseline
	Gr 3 or Gr 4	Permanently discontinue		
Type 1 diabetes mellitus (T1DM) or hyperglycemia	Newly onset T1DM or Grade 3 or 4 hyperglycemia associated with evidence of β-cell failure	Withhold	• Initiate insulin replacement therapy for participants with T1DM<br>• Administer anti-hyperglycemic in participants with hyperglycemia	• Monitor participants for hyperglycemia or other signs and symptoms of diabetes.
Hypophysitis	Gr 2	Withhold	• Administer corticosteroids and initiate hormonal replacements as clinically indicated.	• Monitor for signs and symptoms of hypophysitis (including hypopituitarism and adrenal insufficiency)
	Gr 3 or Gr 4	Withhold or Permanently discontinue		
Hyperthyroidism	Gr 2	Continue	• Treat with non-selective beta-blockers (eg, propranolol) or thionamides as appropriate	• Monitor for signs and symptoms of thyroid disorders.
	Gr 3 or Gr 4	Withhold or permanently discontinue		
Hypothyroidism	Gr 2 – Gr 4	Continue	• Initiate thyroid replacement hormones (eg, levothyroxine or liothyroinine) per standard of care	• Monitor for signs and symptoms of thyroid disorders.
	Gr 1 or Gr 2	Withhold		

Immune-related AEs	Toxicity grade or conditions (CTCAEv4.0)	Action	Event Management	Monitor and Follow-Up
Myocarditis	Gr 3 or Gr 4	Permanently discontinue	• Based on severity of AE administer corticosteroids	• Ensure adequate evaluation to confirm etiology and/or exclude other causes
**Immune-related AEs**	**Toxicity grade or conditions (CTCAEv4.0)**	**Action**	**Event Management**	**Monitor and Follow-Up**
Nephritis and Renal dysfunction	Gr 2	Toxicity resolves to Grade 0-1	Administer corticosteroids (prednisone 1-2 mg/kg or equivalent) followed by taper.	• Monitor changes of renal function
	Gr 3 or Gr 4	Permanently discontinue		
All Other immune-related events	Intolerable or persistent Gr 2	Withhold	Based on type and severity of AE administer corticosteroids	• Ensure adequate evaluation to confirm etiology and/or exclude other causes
	Gr 3	Withhold or discontinue based on the type of event. Events that require discontinuation include and not limited to: Gullain-Barre Syndrome, encephalitis		
	Gr 4 or recurrent Gr 3	Permanently discontinue		

1. Withhold or permanently discontinue pembrolizumab is at the discretion of the investigator or treating physician.

NOTE:
For participants with Grade 3 or 4 immune-related endocrinopathy where withhold of pembrolizumab is required, pembrolizumab may be resumed when AE resolves to ≤ Grade 2 and is controlled with hormonal replacement therapy or achieved metabolic control (in case of T1DM).

Dosing interruptions are permitted in the case of medical/surgical events or logistical reasons not related to study therapy (e.g. Elective surgery, unrelated medical events, patient vacation, and/or holidays). Subjects should be placed on study therapy within 4 weeks of the scheduled interruption. The reason for interruption should be documented in the patient's study record.

5.8 RESCUE MEDICATION AND SUPPORTIVE CARE

5.8.1 Supportive Care Guidelines

Subjects should receive appropriate supportive care measures as deemed necessary by the treating investigator. Suggested supportive care measures for the management of adverse events with potential immunologic etiology are outlined below. Where appropriate, these guidelines include the use of oral or intravenous treatment with corticosteroids as well as additional anti-inflammatory agents if symptoms do not improve with administration of corticosteroids. Note that several courses of steroid tapering may be necessary as symptoms may worsen when the steroid dose is decreased. For each disorder, attempts should be made to rule out other causes such as metastatic disease or bacterial or viral infection, which might require additional supportive care. The treatment guidelines are intended to be applied when the investigator determines the events to be related to either study drug.

Note: if after the evaluation the event is determined not to be related, the investigator does not need to follow the treatment guidance (as outlined below). Refer to Section 5.7 for dose modification.

It may be necessary to perform conditional procedures such as bronchoscopy, endoscopy, or skin photography as part of evaluation of the event.

- **Pneumonitis**:
    - For **Grade 2 events**, treat with systemic corticosteroids. When symptoms improve to Grade 1 or less, steroid taper should be started and continued over no less than 4 weeks.
    - For **Grade 3-4 events**, immediately treat with intravenous steroids. Administer additional anti-inflammatory measures, as needed.
    - Add prophylactic antibiotics for opportunistic infections in the case of prolonged steroid administration.
- **Diarrhea/Colitis**:

  Subjects should be carefully monitored for signs and symptoms of enterocolitis (such as diarrhea, abdominal pain, blood or mucus in stool, with or without fever) and of bowel perforation (such as peritoneal signs and ileus).

    - All subjects who experience diarrhea/colitis should be advised to drink liberal quantities of clear fluids. If sufficient oral fluid intake is not feasible, fluid and

electrolytes should be substituted via IV infusion. For Grade 2 or higher diarrhea, consider GI consultation and endoscopy to confirm or rule out colitis.

- For **Grade 2 diarrhea/colitis,** administer oral corticosteroids.
- For **Grade 3 or 4 diarrhea/colitis,** treat with intravenous steroids followed by high dose oral steroids.
- When symptoms improve to Grade 1 or less, steroid taper should be started and continued over no less than 4 weeks.

- **Type 1 diabetes mellitus (if new onset, including diabetic ketoacidosis [DKA]) or ≥ Grade 3 Hyperglycemia, if associated with ketosis (ketonuria) or metabolic acidosis** (DKA) For **T1DM** or **Grade 3-4** Hyperglycemia
  - Insulin replacement therapy is recommended for Type I diabetes mellitus and for Grade 3-4 hyperglycemia associated with metabolic acidosis or ketonuria.
  - Evaluate patients with serum glucose and a metabolic panel, urine ketones, glycosylated hemoglobin, and C-peptide.
- **Hypophysitis**:
  - For **Grade 2** events, treat with corticosteroids. When symptoms improve to Grade 1 or less, steroid taper should be started and continued over no less than 4 weeks. Replacement of appropriate hormones may be required as the steroid dose is tapered.
  - For **Grade 3-4** events, treat with an initial dose of IV corticosteroids followed by oral corticosteroids. When symptoms improve to Grade 1 or less, steroid taper should be started and continued over no less than 4 weeks. Replacement of appropriate hormones may be required as the steroid dose is tapered.
- **Hyperthyroidism or Hypothyroidism**:

  Thyroid disorders can occur at any time during treatment. Monitor patients for changes in thyroid function (at the start of treatment, periodically during treatment, and as indicated based on clinical evaluation) and for clinical signs and symptoms of thyroid disorders.

  - **Grade 2** hyperthyroidism events (and **Grade 2-4** hypothyroidism):
    - In hyperthyroidism, non-selective beta-blockers (e.g. propranolol) are suggested as initial therapy.
    - In hypothyroidism, thyroid hormone replacement therapy, with levothyroxine or liothyronine, is indicated per standard of care.
  - **Grade 3-4** hyperthyroidism
    - Treat with an initial dose of IV corticosteroid followed by oral corticosteroids. When symptoms improve to Grade 1 or less, steroid taper should be started and continued over no less than 4 weeks. Replacement of appropriate hormones may be required as the steroid dose is tapered.
- **Renal Failure or Nephritis**:
  - For **Grade 2** events, treat with corticosteroids.
  - For **Grade 3-4** events, treat with systemic corticosteroids.
  - When symptoms improve to Grade 1 or less, steroid taper should be started and continued over no less than 4 weeks.
- **Management of Infusion Reactions**:

Signs and symptoms usually develop during or shortly after drug infusion and generally resolve completely within 24 hours of completion of infusion.

Below shows treatment guidelines for subjects who experience an infusion reaction associated with administration of pembrolizumab.

Table 5.3 Infusion Reaction Treatment Guidelines

NCI CTCAE Grade	Treatment	Premedication at subsequent dosing
**<u>Grade 1</u>**<br>Mild reaction; infusion interruption not indicated; intervention not indicated	Increase monitoring of vital signs as medically indicated until the subject is deemed medically stable in the opinion of the investigator.	None
**<u>Grade 2</u>**<br>Requires therapy or infusion interruption but responds promptly to symptomatic treatment (e.g., antihistamines, NSAIDs, narcotics, IV fluids); prophylactic medications indicated for ≤24 hrs	**Stop Infusion.**<br>Additional appropriate medical therapy may include but is not limited to:<br>IV fluids<br>Antihistamines<br>NSAIDs<br>Acetaminophen<br>Narcotics<br><br>Increase monitoring of vital signs as medically indicated until the participant is deemed medically stable in the opinion of the investigator.<br>If symptoms resolve within 1 hour of stopping drug infusion, the infusion may be restarted at 50% of the original infusion rate (e.g. from 100 mL/hr to 50 mL/hr). Otherwise dosing will be held until symptoms resolve and the participant should be premedicated for the next scheduled dose.<br>**Participants who develop Grade 2 toxicity despite adequate premedication should be permanently discontinued from further study drug treatment**	Participant may be premedicated 1.5h (± 30 minutes) prior to infusion of _____ with:<br>Diphenhydramine 50 mg po (or equivalent dose of antihistamine).<br>Acetaminophen 500-1000 mg po (or equivalent dose of analgesic).

**Grade 3:**<br>Prolonged (i.e., not rapidly responsive to symptomatic medication and/or brief interruption of infusion); recurrence of symptoms following initial improvement; hospitalization indicated for other clinical sequelae (e.g., renal impairment, pulmonary infiltrates)<br><br>**Grade 4:**<br>Life-threatening; pressor or ventilatory support indicated	**Stop Infusion.**<br>Additional appropriate medical therapy may include but is not limited to:<br>Epinephrine**<br>IV fluids<br>Antihistamines<br>NSAIDs<br>Acetaminophen<br>Narcotics<br>Oxygen<br>Pressors<br>Corticosteroids<br>Increase monitoring of vital signs as medically indicated until the participant is deemed medically stable in the opinion of the investigator.<br>Hospitalization may be indicated.<br>**In cases of anaphylaxis, epinephrine should be used immediately.<br>**Participant is permanently discontinued from further study drug treatment.**	No subsequent dosing.
Appropriate resuscitation equipment should be available at the bedside and a physician readily available during the period of drug administration.<br>For further information, please refer to the Common Terminology Criteria for Adverse Events v4.0 (CTCAE) at http://ctep.cancer.gov		

5.8.2 Guidance for diagnosis and management of hepatic events of clinical interest

In addition to overdose, hepatic events of clinical interest (ECIs) will include any of the following events. All of these events (in combination) will require holding pembrolizumab and bavituximab treatment, notification of the Sponsor within 24 hours, and a hepatology consultation is strongly recommended. All cases of retreatment and permanent discontinuation must be reported to the Sponsor and recorded in the database.

a. ALT:
   i. Among subjects with Baseline ALT <2×ULN, ECI = ALT ≥5×ULN
   ii. Among subjects with Baseline ALT ≥2×ULN, ECI = ALT >3× the Baseline level
   iii. ALT > 500 U/L regardless of Baseline level

b. AST:
   i. Among subjects with Baseline AST <2×ULN, ECI = AST ≥5×ULN
   ii. Among subjects with Baseline AST ≥2×ULN, ECI = AST >3× the Baseline level
   iii. AST >500 U/L regardless of Baseline level

c. Total Bilirubin:
   i. Among subjects with Baseline levels <1.5 mg/dL, ECI = >2.0 mg/dL
   ii. Among subjects with Baseline levels that are ≥1.5 mg/dL, ECI = ≥2× the Baseline level
   iii. Total bilirubin >3.0 mg/dL regardless of baseline level

d. Regardless of laboratory values, hepatic decompensation diagnosed <u>clinically</u>, including:
   i. New onset clinically detectable ascites
   ii. Gastrointestinal bleeding suggestive of portal hypertension (e.g., esophageal or gastric varices)
   iii. Encephalopathy

Immediate assessment

<u>All subjects</u>

- All subjects should be evaluated according to directions below within 72 hours of alert for non-overdose ECI
- Procedures:
  - Obtain a consultation with a hepatologist
  - Obtain a work-up for hepatitis if there is no underlying hepatitis, including hepatitis A, B,C, D, E, Epstein-Barr virus, and cytomegalovirus

- o Assess for ingestion of drugs/supplements with hepatotoxic potential
- o Assess for alcohol ingestion
- o Assess for potential bacterial infection, biliary obstruction, or occult gastrointestinal bleeding
- o Repeat ALT, AST, Tbil, Dbil, ALP, gamma-glutamyl transpeptidase (GGT), INR, and CBC with differential
- o Other laboratories or imaging studies as clinically indicated o Consider liver biopsy if indicated by hepatologist

Hepatitis C-Infected Subjects (including subjects who previously achieved SVR 12)

- In addition to the above, measure HCV RNA viral load Hepatitis B-infected Subjects
- HBV DNA, HBsAg, HBeAg, anti-HBc (total and IgM), anti-HBe, and anti-HBs
- Subjects should be questioned about compliance with the use of anti-viral agents.

Permanent Discontinuation Criteria for Subjects with Non-overdose Hepatic ECI

Therapy should also be permanently discontinued for any of the following:

- ALT >20×ULN
- CP score of ≥9 points
- Gastrointestinal bleeding suggestive of portal hypertension (e.g., esophageal or gastric varices)
- New onset clinically detectable ascites
- Encephalopathy
- Recurrence of a severe or life-threatening event, or of any of the laboratory abnormalities listed above, that are presumed to be immune-related.

Other subjects may be eligible for treatment interruption (and potential re-start) of pembrolizumab after discussion with the Sponsor.

Diagnosis and Management of Non-Overdose Hepatic ECIs

HCC subjects are at risk for a range of complications that can cause hepatic laboratory abnormalities with or without clinical decompensation. Those with a history of chronic HCV or HBV infection also have the potential to experience virologic flares. Immune-related hepatitis has been observed in ~1% of subjects who received pembrolizumab. The following section provides further guidance on the diagnosis and management of potential hepatic complications among HCC subjects.

a. Hepatitis B Flare

Hepatitis B flares are characterized by rapid elevations of ALT and AST to >5×ULN and/or >3× baseline. ALT elevation to ≥10×ULN is common. In the absence of hepatic decompensation, ALT/AST elevations are typically isolated (i.e., limited/no elevations of bilirubin/ALP). Subjects who are compliant with anti-viral therapy should have continued suppression of HBV DNA at the time of flare; thus, detection of HBV DNA should prompt questioning of subjects for compliance. Laboratory abnormalities secondary to flare are typically observed for 3-5 weeks.

Among subjects with HBV, a flare should be considered if this pattern is observed and there is no evidence of an alternative etiology. Guidelines for subjects with a diagnosis of HBV flare are as follows:

- Care should be instituted in consultation with a hepatologist whenever possible.
- For subjects who have detectable HBV DNA, re-institute anti-viral therapy.
- If the subject is clinically stable, pembrolizumab dosing may be interrupted for up to 12 weeks. Subjects should undergo weekly laboratory tests including: AST, ALT, ALP, Tbil, Dbil, INR, HBsAg, HBV DNA (if detected at the onset of the flare). Obtain anti-HBe, anti-HBs, and HBV DNA levels (if not detected at the onset of the flare) every 23 weeks.
- If ALT returns to normal or Grade 1 (if normal at baseline), or to baseline grade (if Grade 2 at baseline) within 12 weeks, and subjects are clinically stable, subjects may restart pembrolizumab treatment. If these conditions are not met, then pembrolizumab treatment should be permanently discontinued.

b. Hepatitis C Recurrence or Flare

Subjects who achieved SVR 12 and subjects with ongoing HCV infection are eligible for enrollment. In rare circumstances, HCV subjects who achieve SVR 12 may relapse at later time points. Relapse is characterized by detection of HCV RNA, often accompanied by ALT elevations to >5×ULN. In the absence of hepatic decompensation, ALT/AST elevations are typically isolated (i.e., limited/no elevations of bilirubin/ALP).

Among subjects with uncontrolled hepatitis C, virologic flares are possible. Hepatitis C flares are characterized by rapid elevations of ALT and AST to >5×ULN and/or >3× baseline along with a rise in HCV RNA. ALT elevation to ≥10×ULN and a 1 log elevation in HCV RNA level are common. In the absence of hepatic decompensation, ALT/AST elevations are typically isolated (i.e., limited/no elevations of bilirubin/ALP). Laboratory abnormalities secondary to flare or recurrence are typically observed for 3-5 weeks.

Guidelines for subjects with recurrent HCV infection or an HCV flare are described below:

i. Recurrent HCV infection:

If the subject entered the study with an HCV RNA test of “Target not Detected” and has confirmed detectable HCV RNA (2 specimens, 1 week apart), then the subject has experienced a late HCV relapse or a recurrent infection. Question the subject about use of injection or inhalation drugs

- At the time of first detection of HCV RNA, send a specimen for HCV genotyping
- Measure AST, ALT, ALP, Tbil, Dbil, and INR weekly
- Measure HCV RNA levels every 2 weeks
- Therapy with HCV anti-viral treatments should be strongly considered.

ii. HCV Flare:

- At the time of first detection of HCV RNA, send a specimen for HCV genotyping

- Measure AST, ALT, ALP, Tbil, Dbil, INR weekly
- Measure HCV RNA levels every 2 weeks
- Therapy with HCV anti-viral treatments should be strongly considered.

iii. For both recurrent infection and HCV flare: if ALT returns to normal or Grade 1 (if normal at baseline), or to baseline grade (if Grade 2 at baseline) within 12 weeks, and the subjects are clinically stable, subjects may restart pembrolizumab treatment. If these conditions are not met, then pembrolizumab treatment should be permanently discontinued.

c. Immune-related hepatitis

i. Description: Immune-related hepatitis due to pembrolizumab should be suspected if any of the following is seen:

- AST or ALT baseline values are less than 2×ULN, and AST or ALT laboratory values increase to ≥5×ULN
- Among subjects with Day 1 ALT or AST ≥2×ULN, levels increase to >3× the Day 1 level
- AST/ALT >500 U/L regardless of baseline level
- Among subjects with Day 1 Tbil levels <1.5 mg/dL: a value of >2.0 mg/dL
- Among subjects with Day 1 Tbil levels that are ≥1.5 mg/dL: a value of ≥2× the Day 1 level
- Total bilirubin >3.0 mg/dL regardless of baseline level.

Immune-related hepatitis is a diagnosis made after excluding other possible etiologies for the change. Viral flare (if applicable), biliary or vascular obstruction, infection, medications, and alcohol use must be ruled out (see below).

ii. Management

- Interrupt pembrolizumab treatment and alert the sponsor as per ECI criteria above for ALT, AST, bilirubin, and hepatic decompensation.
- Start IV corticosteroid (methylprednisolone 125 mg) followed by oral corticosteroid (1-2 mg/kg/day).
- Monitor with biweekly laboratory tests including AST, ALT, Tbil, Dbil, ALP, and INR.
- If symptoms and laboratory tests resolve to Grade ≤1 or baseline (if abnormal at baseline), taper steroids over 28 days. Pembrolizumab treatment may be restarted after steroid treatment has been tapered to prednisone ≤10 mg/day (or equivalent dose of another agent). Treatment and laboratory results must be reported on a CRF.
- If laboratory abnormalities do not resolve within 3 weeks, or steroids cannot be lowered to ≤10 mg/day (or prednisone equivalent) within 12 weeks, or subjects show evidence of decompensation to CP C status or have esophageal or variceal bleeding at any point, treatment must be permanently discontinued. This must be reported on a CRF.

d. Other Hepatic Events of Clinical Interest

- Infection needs to be ruled out with cultures of blood, urine, and ascites (if possible), as well as chest x-ray and abdominal imaging if relevant. If an infection is found, antibiotics should be started.

- If Tbil is elevated above baseline, magnetic resonance cholangiopancreatography or ultrasound with doppler should be obtained to rule out vascular compromise, biliary obstruction, and/or tumor progression. If biliary obstruction is present, consultation with a gastroenterologist and/or an interventional radiologist should be obtained to see if the obstruction may be relieved.
- A careful review of drugs, including herbal and alternative medications, should be obtained, and alcohol use should be ruled out. See Section 5.5.2 for drugs which may interfere with hepatic function.
- For all of these cases, subjects may resume pembrolizumab treatment if they are clinically stable after appropriate therapy or discontinue the causative agent, as long as laboratory values have returned to Grade 1 or baseline (if normal or Grade 1 at start) or to baseline grade within 3 weeks.
- Treatment must be permanently discontinued if the subject is off pembrolizumab therapy for infection, obstruction, or drug/alcohol-related toxicity for more than 3 weeks, or if they have esophageal bleeding, or become CP C at any point.

5.9 CONTRACEPTION

Pembrolizumab and bavituximab may have adverse effects on a fetus in utero.
Furthermore, it is not known if pembrolizumab or bavituximab has transient adverse effects on the composition of sperm.

For this trial, male subjects will be considered to be of non-reproductive potential if they have azoospermia (whether due to having had a vasectomy or due to an underlying medical condition).

Female subjects will be considered of non-reproductive potential if they are either:

(1) postmenopausal (defined as at least 12 months with no menses without an alternative medical cause; in women < 45 years of age a high follicle stimulating hormone (FSH) level in the postmenopausal range may be used to confirm a post-menopausal state in women not using hormonal contraception or hormonal replacement therapy. In the absence of 12 months of amenorrhea, a single FSH measurement is insufficient.); OR

(2) have had a hysterectomy and/or bilateral oophorectomy, bilateral salpingectomy or bilateral tubal ligation/occlusion, at least 6 weeks prior to screening; OR

(3) has a congenital or acquired condition that prevents childbearing.

Female and male subjects of reproductive potential must agree to avoid becoming pregnant or impregnating a partner, respectively, while receiving study drug and for 120 days after the last dose of study drug by complying with one of the following:

(1) practice abstinence† from heterosexual activity; OR

(2) use (or have their partner use) acceptable contraception during heterosexual activity.

Acceptable methods of contraception are‡:

Single method (one of the following is acceptable):

- intrauterine device (IUD)
- vasectomy of a female subject's male partner
- contraceptive rod implanted into the skin

Combination method (requires use of two of the following):

- diaphragm with spermicide (cannot be used in conjunction with cervical cap/spermicide)
- cervical cap with spermicide (nulliparous women only)
- contraceptive sponge (nulliparous women only)
- male condom or female condom (cannot be used together)
- hormonal contraceptive: oral contraceptive pill (estrogen/progestin pill or progestin only pill), contraceptive skin patch, vaginal contraceptive ring, or subcutaneous contraceptive injection

†Abstinence (relative to heterosexual activity) can be used as the sole method of contraception if it is consistently employed as the subject's preferred and usual lifestyle and if considered acceptable by local regulatory agencies and ERCs/IRBs. Periodic abstinence (e.g., calendar, ovulation, sympto-thermal, post-ovulation methods, etc.) and withdrawal are not acceptable methods of contraception.

‡If a contraceptive method listed above is restricted by local regulations/guidelines, then it does not qualify as an acceptable method of contraception for subjects participating at sites in this country/region.

Subjects should be informed that taking the study medication may involve unknown risks to the fetus (unborn baby) if pregnancy were to occur during the study. In order to participate in the study subjects of childbearing potential must adhere to the contraception requirement (described above) from the day of study medication initiation) throughout the study period, and through 120 days after the last dose of trial therapy. If there is any question that a subject of childbearing potential will not reliably comply with the requirements for contraception, that subject should not be entered into the study.

5.9.1 Use in Pregnancy

If a subject inadvertently becomes pregnant while on treatment with pembrolizumab or bavituximab, the subject will immediately be removed from the study. The site will contact the subject at least monthly and document the subject's status until the pregnancy has been completed or terminated. The outcome of the pregnancy will be reported to Merck without delay and within 24 hours to Merck if the outcome is a serious adverse experience (e.g., death, abortion, congenital anomaly, or other disabling or life-threatening complication to the mother or newborn).

The study investigator will make every effort to obtain permission to follow the outcome of the pregnancy and report the condition of the fetus or newborn to the Sponsor. If

a male subject impregnates his female partner the study personnel at the site must be informed immediately and the pregnancy reported to Merck and followed as described above.

5.9.2 Use in Nursing Women

It is unknown whether pembrolizumab or bavituximab is excreted in human milk. Since many drugs are excreted in human milk, and because of the potential for serious adverse reactions in the nursing infant, subjects who are breast-feeding are not eligible for enrollment.

5.10 DRUG SUPPLY AND STORAGE

Study drug must be received by the designated personnel at the study site, handled and stored safely and properly, and kept in a secured location to which only designated personnel have access. Upon receipt, the study drug should be stored according to the instructions specified on the drug labels and in the Investigators Brochure. Medication labels will comply with the legal requirements of each country and be printed in the local language.

6.0 STUDY PROCEDURES

6.1 SCREENING PHASE

Assessments performed exclusively to determine eligibility for this study will be done only after obtaining informed consent. Assessments performed for clinical indications (not exclusively to determine study eligibility) may be used for baseline values even if the studies were done before informed consent was obtained.

All screening procedures must be performed within 28 days prior to first treatment (Cycle 1, Day 1), unless otherwise stated. The screening procedures include the following: (See Table 6.1 above for the list of assessments to be performed.)

6.1.1 Informed Consent

Patients must provide a signed informed consent form prior to any study specific evaluations including screening. Eligibility will be determined according to the inclusion/exclusion criteria as described in Section 4. A list of procedures to be performed at time of screening is summarized in Table 6.1. Patients must meet all eligibility criteria to be considered for enrollment in the study.

6.1.2 Eligibility Checklist (Inclusion/Exclusion)

In order to determine and confirm the eligibility of the patient, once all screening procedures are completed.

6.1.3 Patient Demographics and other Baseline Characteristics

The data that will be collected on patient characteristics at screening includes:

- Demographic (name, date of birth, age), sex, race/ethnicity;
- Diagnosis, and extent of cancer (staging);
- Pertinent Medical History;
- Prior cancer treatment;
- All medications taken within 30 days before first treatment. If there are any changes, medications are to be updated on a continual basis.

Furthermore the following assessments will be performed:

- Vital Signs (BP, HR, T, RR);
- Adverse Events / Concomitant medications;
- Height and Weight;
- Physical Examination (PE);
- ECOG Performance Status (PS);
- Child-Pugh Score
- Laboratory Evaluations (CMP, CBC, PT/INR and aPTT, thyroid panel, AFP, Hepatitis B and C panel, and UA)
- ECG
- Collection of your tissue from a previous biopsy or surgery
- Radiological Assessments (RECIST 1.1) (CT - chest/abdomen/pelvis or MRI – abdomen/pelvis). Chest CT will be performed at baseline only. During the treatment phase, a Chest CT will be performed as clinically indicated;
- Urine or serum Pregnancy Test (Women of Child-bearing Potential Only).

6.1.4 Pre-Treatment - Tissue Biopsy

Unless the patient has archival formalin fixed paraffin embedded tissue to document a diagnosis of HCC, a biopsy confirming HCC diagnosis must be completed within 28 days prior to Cycle 1 Day 1.

6.1.5 Biomarker / DNA Blood Collection (Optional)

After signing the optional biomarker / DNA consent for future biomarker and DNA blood, samples will be collected at baseline, during treatment, and at the end of treatment or early-term visit. This procedure is for research and will not be used to make medical decisions regarding the patient's treatment.

6.2 TREATMENT PHASE

6.2.1 Study Treatment

Patients will be treated with pembrolizumab with 200 mg intravenously once every 3 weeks (for a maximum of 35 treatments). In addition, patients will be treated with bavituximab (3.0 mg/kg intravenously weekly) until disease progression, unacceptable toxicity, death, or discontinuation from the study treatment due to any other reason. For details of assessment, refer to Table 6.1.

If the patient is unable to receive treatment on D1 for any other reason than the listed dosing parameters in section 5, the entire cycle will be delayed no longer than 6 weeks.

6.2.2 Post-Treatment – Biomarker Tissue Biopsy (Optional)

The post treatment biopsy is optional and will be performed for patients who consented to the procedure during the initial consenting process. Following the initial treatment of study drug, one correlative biopsy will be performed between the 3rd and 4th week of treatment. Most accessible site will be biopsied based on investigators judgement and performed with imaging guidance, as indicated. Tissue will be processed using institutional protocols. Tissue from sub sites will be shipped to UTSW. Samples will be analyzed in batches during and after enrollment.

6.3 POST – TREATMENT FOLLOW-UP PHASE

6.3.1 End of Treatment

Patients who completely discontinue study treatment should complete an End of Treatment (EOT) visit at the time of discontinuation. For details of the assessment, see Table 6-1.

At a minimum, all patients who discontinue study treatment, including those who refuse to return for a final visit, will be contacted for study evaluations during the 30 days following the last dose date of study treatment.

6.3.1.1 Criteria for Early Withdrawal

Patients may voluntarily withdraw from the study treatment or be taken off study at the discretion of the investigator at any time. Patients may be withdrawn from the study treatment if any of the following occur:

- Adverse event
- Lost to follow-up
- Physician decision
- 2nd occurrence of progressive disease
- Protocol deviation
- Study terminated by the sponsor
- Technical problems

6.3.2 Safety Follow-Up

All patients will be followed for safety up to 30 days after last dose of Bavituximab treatment. Patients whose treatment is interrupted or permanently discontinued due to an AE, including abnormal laboratory value, must be followed until resolution or stabilization of the event, whichever comes first.

Serious adverse events that occur within 90 days (or 30 days if a new anti-cancer therapy is started) of the End of Treatment visit should be followed and recorded, regardless of drug relationship.

6.3.3 Survival Follow-Up

All patients will be followed for survival status every 12 weeks, starting from the Safety Follow-Up visit, regardless of treatment discontinuation, until death, lost to follow-up, or withdrawal of consent to survival follow-up.

Survival follow-up assessment may be made by a phone call to the patient or designated PHI, EPIC medical record review, or certified mail. At least 3 documented attempts should be made to contact the patient before the patient is considered lost to follow-up.

6.3.4 Beginning and End of the Trial

The study begins when the first patient signs the informed consent. The end of the study may be designated as the time point when all patients have discontinued the

study or are a minimum of 6 months post-study medication. At this point, a database lock of the trial may occur to allow the analysis of the study data. Any remaining patients may continue to receive study medication and be seen by the investigator per usual standard of care for this patient population.

Trial Period:	Screen	Treatment						End of Treatment	Follow- up	
Treatment Cycle/Title:	Screen	C1D1	C1D8	C1D15	C2D1+	C2D8+	C2D15+	Time of Discontinuation	Safety Follow-up	Survival Follow-up[a]
									30 Days Post Last Dose	
**Scheduling Window (Days)**	-28 to -1		±3	±3	±3	±3	±3	±3	±7	±7
**Administrative Procedures**										
Informed consent	X									
Optional consent for future research	X									
Inclusion/exclusion criteria	X									
Demographics and medical history	X									
Prior and concomitant medication review	X	X	X	X	X	X	X	X	X	
Post-study anticancer therapy status									X	X
Survival status										X
**Clinical Procedures/Assessments**										
Review adverse events	X	X	X	X	X	X	X	X	X	
History and Physical examination	X	X		X	X					
Child-Pugh Score	X									
Height, weight, and vital signs (T, HR, RR, BP) [c]	X	X	X	X	X	X	X	X		
12-Lead electrocardiogram	X									
ECOG performance status	X	X		X	X			X		
Trial treatment administration[d]		X [b]	X	X	X	X	X			
**LOCAL Laboratory Assessments**										
Pregnancy test	X									
PT/INR and aPTT	X	X						X	X	

Trial Period:	Screen	Treatment						End of Treatment	Follow- up	
Treatment Cycle/Title:	Screen	C1D1	C1D8	C1D15	C2D1+	C2D8+	C2D15+	Discontinuation	Safety Follow-up	Survival Follow-up[a]
									30 Days Post Last Dose[i]	
Scheduling Window (Days)	-28 to -1		±3	±3	±3	±3	±3	±3	±7	±7
**LOCAL Laboratory Assessments**										
CBC with differential	X	X	X	X	X	X	X	X	X	
Chemistry Panel	X	X	X	X	X	X	X	X	X	
Urinalysis	X				X[i]			X		
FT3, FT4, TSH	X				X[i]			X	X	
AFP	X				X			X	X	
Anti-HCV	X									
**If Anti-HCV positive:**										
HCV genotype	X									
HCV viral load, HBsAg, HBV viral load[e]	X				X				X	
Anti-HBc (total and IgM), anti-HBs	X									
**If HbsAg+ or anti-HBc+, anti-HBs-, HbsAg- and viral load <100 IU/mL:**										
Anti-HDV	X									
HBeAg and anti-HBe	X				X[f]				X	
Anti-HBc (total) and anti-HBs					X[f]				X	
Research Assessments										
Biopsy: Tumor tissue for biomarker analysis[g]	X				X[g]					
Archived Tumor Tissue Collection	X									
Tumor assessment by RECIST 1.1	X	Q 9 weeks for the 1st 54 weeks and Q 12 weeks until PD						X[h]		

a. In subjects that experience PD or start a new anti-cancer therapy, contact should be made (by telephone, medical record review or visit) Q12W from Safety Follow-up Visit to assess for survival status.

b. Screening labs can be used for C1W1 if performed within 72 hours.

c. Height will be measured at Visit 1 only.

d. Treatment will consist of Bavituximab administered weekly and Pembrolizumab administered every three weeks; cycles will consist of three week long periods with Pembrolizumab constituting the beginning of a new cycle.

e. Can be performed earlier if clinically indicated.

f. Performed every 12 weeks during treatment, or earlier if clinically indicated. Additional tests to be performed for ECIs are described in Section 5.2.1.38.2. If the viral load results are not reported in IU/mL, the site should provide the conversion factor specific for the assay method used to convert the assay units of measure to IU/mL. For HbsAg, quantitative and/or qualitative results are submitted.

g. Biomarker blood collection is an optional research procedure and patients must have consented to the additional blood collection to participate. Blood will be collected according to the schedule in the table.

h. The biopsy performed during screening for HCC diagnosis and must be performed for the subject to be eligible for the study. If the patient has archival formalin fixed paraffin embedded tissue no additional baseline biopsy will be done. The post-treatment biopsy is optional and for research purposes only. If the subject consents to the research biopsy during the consent process, it will be performed during the 3rd and 4th week after initiating Bavituximab/Pembrolizumab therapy.

i. Completed on Day 1 of even cycles, beginning with Cycle 2.

j. Only required if discontinued for any reason other than PD. Continue with tumor assessments per schedule until radiographic progression.

k. 30 days post last dose of Bavituximab

.

7.0 MEASUREMENT OF EFFECT

For the purposes of this study, patients will be evaluated for progression every 9 weeks for the first 54 weeks, then every 12 weeks thereafter following the initial study drug (pembrolizumab and bavituximab) administration. Imaging will consist of a CT or MRI abdomen with liver protocol. The same imaging modality used at baseline will be used for further scans unless documented by primary investigator. CT Chest or additional imaging will be at the discretion of the investigator based on clinical suspicion of additional progressive disease.

7.1 ANTITUMOR EFFECT- SOLID TUMORS

Response and progression will be evaluated in this study using the new international criteria proposed by the Response Evaluation Criteria in Solid Tumors (RECIST) Committee [JNCI 92(3):205-216, 2000]. Changes in only the largest diameter (unidimensional measurement) of the tumor lesions are used in the RECIST 1.1 criteria. Note: Lesions are either measurable or non-measurable using the criteria provided below. The term “evaluable” in

reference to measurability will not be used because it does not provide additional meaning or accuracy.

7.1.1 Disease Parameters

Measurable Disease. Measurable lesions are defined as those that can be accurately measured in at least one dimension (longest diameter to be recorded) as ≥ 20 mm with conventional techniques (CT, MRI, x-ray) or as ≥ 10 mm with spiral CT scan. All tumor measurements must be recorded in millimeters (or decimal fractions of centimeters). A “high-resolution” CT scan is one in which images are recorded at least every 5 mm.

Non-Measurable Disease. All other lesions (or sites of disease), including small lesions (longest diameter <20 mm with conventional techniques or <10 mm using spiral CT scan), are considered non-measurable disease. Bone lesions, leptomeningeal disease, ascites, pleural/pericardial effusions, lymphangitis cutis/pulmonis, inflammatory breast disease, abdominal masses (not followed by CT or MRI), and cystic lesions are all non-measurable.

Target Lesions. All measurable lesions up to a maximum of 2 lesions per organ and 5 lesions in total, representative of all involved organs, should be identified as **target lesions** and recorded and measured at baseline. Target lesions should be selected on the basis of their size (lesions with the longest diameter) and their suitability for accurate repeated measurements (either by imaging techniques or clinically). A sum of the longest diameter (LD) for all target lesions will be calculated and reported as the baseline sum LD. The baseline sum LD will be used as reference by which to characterize the objective tumor response.

Non-Target Lesions. All other lesions (or sites of disease) that are not target lesions, as defined in the section above, will be identified as non-target lesions and should also be recorded at baseline. Non-target lesions include measurable lesions that exceed the maximum numbers per organ or total of all involved organs as well as non-measurable lesions. Measurements of these lesions are required when feasible, since a patient may have progressive disease on the basis of larger non-target lesions. The presence or absence of each non-target lesion should be noted throughout follow-up.

7.1.2 Methods for Evaluation of Measurable Disease

All measurements should be taken and recorded in metric notation using a ruler or calipers. All baseline evaluations should be performed as closely as possible to the beginning of treatment and never more than 28 days before the beginning of the treatment.

The same method of assessment and the same technique should be used to characterize each identified and reported lesion at baseline and during follow-up. Imaging-based evaluation is preferred to evaluation by clinical examination when both methods have been used to assess the antitumor effect of a treatment.

7.1.3 Response Criteria

7.1.3.1 Evaluation of Target Lesions

Complete Response (CR): Disappearance of all target lesions, determined by two separate observations conducted not less than 4 weeks apart. There can be no appearance of new lesions.

Partial Response (PR): At least a 30% decrease in the sum of the longest diameter (LD) of target lesions, taking as reference the baseline sum LD. There can be no appearance of new lesions.

Progressive Disease (PD): At least a 20% increase in the sum of the LD of target lesions, taking as reference the smallest sum LD recorded since the treatment started, or the appearance of one or more new lesions.

Stable Disease (SD): Neither sufficient shrinkage to qualify for PR nor sufficient increase to qualify for PD, taking as reference the smallest sum LD since the treatment started.

7.1.3.2 Evaluation of Non-Target Lesions

Complete Response (CR): Disappearance of all non-target lesions and normalization of tumor marker level.

Incomplete Response/Stable Disease (SD): Persistence of one or more non-target lesion(s) and/or maintenance of tumor marker level above the normal limits.

Progressive Disease (PD): Appearance of one or more new lesions and/or unequivocal progression of existing non-target lesions

7.1.3.3 Evaluation of Best Overall Response

The best overall response is the best response recorded from the start of the treatment until disease progression/recurrence (taking as reference for progressive disease the smallest measurements recorded since the treatment started). The patient's best response assignment will depend on the achievement of both measurement and confirmation criteria.

**Target Lesions**	**Non-Target Lesions**	**New Lesions**	**Overall Response**	**Best Response for this Category Also Requires:**
CR	CR	No	CR	≥4 wks. confirmation

CR	Non-CR/Non-PD	No	PR	>4 wks.
PR	Non-PD	No	PR	confirmation
SD	Non-PD	No	SD	documented at least once >4 wks. from baseline
PD	Any	Yes or No	PD	no prior SD, PR or CR
Any	PD*	Yes or No	PD	
Any	Any	Yes	PD	

* In exceptional circumstances, unequivocal progression in non-target lesions may be accepted as disease progression.

Note: Subjects with a global deterioration of health status requiring discontinuation of treatment without objective evidence of disease progression at that time should be reported as "*symptomatic deterioration*". Every effort should be made to document the objective progression even after discontinuation of treatment.

Note: If subjects respond to treatment and are able to have their disease resected, the patient's response will be assessed prior to the surgery.

7.1.4 Duration of Response

Duration of overall response: The duration of overall response is measured from the time measurement criteria are met for CR or PR (whichever is first recorded) until the first date that recurrent or progressive disease is objectively documented (taking as reference for progressive disease the smallest measurements recorded since the treatment started).

The duration of overall CR is measured from the time measurement criteria are first met for CR until the first date that recurrent disease is objectively documented.

Duration of stable disease: Stable disease is measured from the start of the treatment until the criteria for progression are met, taking as reference the smallest measurements recorded since the treatment started.

7.1.5 Progression free survival

Progression free survival (PFS) is defined as the duration of time from start of drug treatment (pembrolizumab and bavituximab) to time of progression. Deaths during the study period will be included as an event.

7.2 TREATMENT BEYOND PROGRESSION

Accumulating clinic evidence indicates some subjects treated with immune system stimulating agents may develop disease progression by conventional response criteria before demonstrating clinical objective responses and/or stable disease (see section 1.6). This study was observed in the phase Ib study of pembrolizumab, KEYNOTE -001, and confirmed in

multiple phase 3 clinical trials using PD-1 inhibitors. Two hypotheses explain this phenomenon. First, enhanced inflammation within tumor could lead to an increase in tumor size, which would appear as enlarged index lesions and as newly visible small non-index lesion. Over time, both malignant and inflammatory portions of the mass may then decrease, leading to overt signs of clinical improvement. Alternatively, in some individuals, the kinetics of tumor growth may initially outpace anti-tumor immune activity. With sufficient time, the anti-tumor activity will dominate and become clinically apparent.

Therefore, subjects should continue study therapy after an initial investigator-assessed RECIST 1.1-defined progression (section 7.1) as long as they meet the following criteria:

- Investigator assessed clinical benefit, and
- Subject is tolerating the study drug (pembrolizumab and bavituximab)

Subjects should discontinue study therapy upon evidence of further progression, defined as an additional 10% or greater increase in tumor burden from time of initial progression (including all target lesions and new measurable lesions). New lesions are considered measurable if the longest diameter is at least 10 mm (except for pathological lymph nodes, which must have a short axis of at least 15 mm). Any new lesion considered non-measurable may become measurable and therefore included in the tumor burden measurement if the longest diameter increases to at least 10 mm (except for pathological lymph nodes, which must have an increase in short axis to at least 15 mm).

For statistical analyses that include the investigator-assessed progression date, subjects who continue treatment beyond initial investigator-assessed RECIST 1.1 defined progression will be considered to have progressive disease at the time of the initial progression event irrespective of confirmation on subsequent imaging.

7.3 SAFETY/TOLERABILITY

Analyses will be performed for all subjects having received at least one dose of study drug. The study will use the CTCAE version 4.03 for reporting of adverse events (Common Terminology Criteria for Adverse Events (CTCAE) (nih.gov)).

8.0 ADVERSE EVENTS

8.1 PEMBROLIZUMAB TREATMENT RELATED EVENTS

The following side effects are suspected to be related to pembrolizumab treatment for reporting purposes:

>20% of patients

- Itching of the skin
- Loose or watery stools
- Cough

10-20% of patients

- Joint pain
- Fever
- Back pain

- Rash

1 to 10% of patients

- Hypo- or hyperthyroidism
- Inflammation in the lungs
- Inflammation in the colon
- Inflammation in the skin
- Abdominal pain
- Loss of skin color
- Hyponatremia
- Hypotension, flushing, rash, fever, shortness of breath or abdominal pain at the time of the infusion or just after

8.2 BAVITUXIMAB TREATMENT RELATED EVENTS

The following side effects are suspected to be related to bavituximab treatment for reporting purposes:

- Infusion reaction
- Thromboembolic events
- Nausea, vomiting, anorexia
- Prolonged coagulation times

8.3 ADVERSE EVENT MONITORING

Adverse event data collection and reporting, which are required as part of every clinical trial, are done to ensure the safety of subjects enrolled in the studies as well as those who will enroll in future studies using similar agents. Adverse events are reported in a routine manner at scheduled times during a trial. Additionally, certain adverse events must be reported in an expedited manner to allow for optimal monitoring of subject safety and care.

All subjects experiencing an adverse event, regardless of its relationship to study drug, will be monitored until:

- the adverse event resolves or the symptoms or signs that constitute the adverse event return to baseline;
- any abnormal laboratory values have returned to baseline;
- there is a satisfactory explanation other than the study drug for the changes observed; or death.

8.3.1 Definition

An adverse event is defined as any untoward or unfavorable medical occurrence in a human research study participant, including any abnormal sign (for example, abnormal physical exam or clinically significant laboratory finding), symptom, clinical event, or disease, temporarily associated with the subject’s participation in the research, whether or not it is considered related to the subject’s participation in the research.

Adverse events encompass clinical, physical and psychological harms. Adverse events occur most commonly in the context of biomedical research, although on occasion, they can occur in the context of social and behavioral research. Adverse events may be expected or unexpected.

Severity

Adverse events will be graded by a numerical score according to the defined NCI Common Terminology Criteria for Adverse Events (NCI CTCAE v 4.0). Adverse events not specifically defined in the NCI CTCAE will be scored on the Adverse Event log according to the general guidelines provided by the NCI CTCAE and as outlined below.

- Grade 1: Mild;
- Grade 2: Moderate;
- Grade 3: Severe or medically significant but not immediately life threatening;
- Grade 4: Life threatening consequences;
- Grade 5: Death related to the adverse event

Serious Adverse Events

ICH Guideline E2A and the UTSW IRB define serious adverse events as those events, occurring at any dose, which meets any of the following criteria:

- Results in death;
- Immediately life-threatening;
- Results in inpatient hospitalization or prolongation of existing hospitalization;
- Results in persistent or significant disability/incapacity;
- Results in a congenital anomaly/birth defect;
- Based upon appropriate medical judgment, may jeopardize the subject’s health and may require medical or surgical intervention to prevent one of the other outcomes listed in this definition.

Note: A “Serious adverse event” is by definition an event that meets any of the above criteria. Serious adverse events may or may not be related to the research project. A serious adverse event determination does not require the event to be related to the research. That is, both events completely unrelated to the condition under study and events that are expected in the context of the condition under study may be serious adverse events, independent of relatedness to the study itself. As examples, a car accident requiring overnight hospitalization would be a serious adverse event for any research participant; likewise, in a study investigating end-stage cancer care, any hospitalization or death would be a serious adverse event, even if the event observed is a primary clinical endpoint of the study.

Refer to the UTSW IRB website at http://www.utsouthwestern.net/intranet/research/research-administration/irb/studymanagement/adverse-events.html to determine when a serious adverse event requires reporting to the IRB.

8.3.2 Unanticipated Problems Involving Risks to Subjects or Others (UPIRSOs):

The phrase “unanticipated problems involving risks to subjects or others” is found, but not defined in the HHS regulations at 45 CFR 46, and the FDA regulations at 21 CFR 56.108(b)(1) and 21 CFR 312.66. For device studies, part 812 uses the term unanticipated adverse device effect, which is defined in 21 CFR 812.3(s). Guidance from the regulatory agencies considers unanticipated problems to include any incident, experience, or outcome that meets ALL three (3) of the following criteria:

- Unexpected in terms of nature, severity or frequency given (a) the research procedures that are described in the protocol-related documents, such as the IRB-approved research protocol and informed consent document; and (b) the characteristics of the subject population being studied;

  AND

- Related or possibly related to participation in the research (possibly related means there is a reasonable possibility that the incident, experience, or outcome may have been caused by the procedures involved in the research);

  AND

- Suggests that the research places subjects or others at greater risk of harm (including physical, psychological, economic, or social harm) than was previously known or recognized. Note: According to OHRP, if the adverse event is serious, it would always suggest a greater risk of harm.

Follow-up

All adverse events will be followed up according to good medical practices.

8.3.3 Steps to Determine If a Serious Adverse Event Requires Expedited Reporting to the SCCC DSMC

Step 1: Identify the type of adverse event using the NCI Common Terminology Criteria for Adverse Events (CTCAE v5).

Step 2: Grade the adverse event using the NCI CTCAE v5.

Step 3: Determine whether the adverse event is related to the protocol therapy.

Attribution categories are as follows:

- Definite – The AE *is clearly related* to the study treatment.
- Probable – The AE *is likely related* to the study treatment.
- Possible – The AE *may be related* to the study treatment.
- Unlikely – The AE *may NOT be related* to the study treatment.
- Unrelated – The AE *is clearly NOT related* to the study treatment.

Note: This includes all events that occur within 30 days of the last dose of protocol treatment *(alternatively, to the end of the acute adverse events reporting period as defined in section XX)*. Any event that occurs more than 30 days after the last dose of treatment *(alternatively, during the late adverse event period as defined in section XX)* and is attributed (possibly, probably, or definitely) to the agent(s) must also be reported as indicated in the sections below.

Step 4: Determine the prior experience of the adverse event. Expected events are those that have been previously identified as resulting from administration of the treatment. An adverse event is considered unexpected, for expedited reporting purposes only, when either the type of event or the severity of the event is not listed in:

- the current known adverse events listed in the Agent Information Section of this protocol (if applicable);
- the drug package insert (if applicable);
- the current Investigator's Brochure (if applicable)
- the Study Agent(s)/Therapy(ies) Background and Associated Known Toxicities section of this protocol

8.3.4 Reporting SAEs and UPIRSOs

8.3.4.1 Reporting SAEs and UPIRSOs to the Simmons Comprehensive Cancer Center (SCCC) Data Safety Monitoring Committee (DSMC)

SAEs and UPIRSOs at all sites, which occur in research subjects on protocols for which the SCCC is the DSMC of record require reporting to the DSMC regardless of whether IRB reporting is required. All SAEs occurring during the protocol-specified monitoring period and all UPIRSOs should be submitted to the SCCC DSMC within 5 business days of the study team members awareness of the event(s). In addition, for participating centers other than UTSW, local IRB guidance should be followed for local reporting of serious adverse events or unanticipated problems.

The UTSW study PI is responsible for ensuring SAEs/UPIRSOs are submitted to the SCCC DSMC Coordinator. This may be facilitated by the IIT project manager, study team, sub-site or other designee. Hardcopies or electronic versions of the eIRB Reportable Event report; FDA Form #3500A forms, or other sponsor forms, if applicable; and/or any other supporting documentation available should be submitted to the DSMC Coordinator. The DSMC Coordinator forwards the information onto the DSMC Chairman who determines if immediate action is required. Follow-up eIRB reports, and all subsequent SAE or UPIRSO documentation that is available are also submitted to the DSMC Chair who determines if further action is required. *(See Appendix III of the SCCC DSMC Plan for a template Serious Adverse Event Form which may be utilized).*

If the event occurs on a multi-institutional clinical trial coordinated by the UTSW Simmons Comprehensive Cancer Center, the IIT Project Manager or designee ensures that all participating sites are notified of the event and resulting action, according to FDA guidance for expedited reporting. DSMC Chairperson reviews all SAEs and UPIRSOs upon receipt from the DSMC Coordinator. The DSMC Chairperson determines whether action is required and either takes action immediately, convenes a special DSMC session (physical or electronic), or defers the action until a regularly scheduled DSMC meeting.

Written reports to:<br><br>David Hsieh, MD<br>c/o GI Clinical Research Manager<br>5323 Harry Hines Blvd, NB2.418<br>Dallas, Texas 75390<br>Phone: 214-648-7031<br>Fax: 214-648-1906<br>Email: David.hsieh@utsouthwestern.edu<br><br>UTSW SCCC Data Safety Monitoring Committee Coordinator<br>Email: SCCCDSMC@utsouthwestern.edu<br>Fax: 214-648-5949 or deliver to BLB.306<br><br>UTSW Institutional Review Board (IRB)<br>Submit via eIRB with a copy of the final sponsor report as attached supporting documentation.

8.3.4.2 Reporting Unanticipated Problems Involving Risks to Subjects or Others (UPIRSOs) to the UTSW HRPP

UTSW reportable event guidance applies to all research conducted by or on behalf of UT Southwestern, its affiliates, and investigators, sites, or institutions relying on the UT Southwestern IRB. Additional reporting requirements apply for research relying on a non-UT Southwestern IRB.

According to UTSW HRPP policy, UPIRSOs are incidents, experiences, outcomes, etc. that meet **ALL three (3)** of the following criteria:

1. Unexpected in nature, frequency, or severity (i.e., generally not expected in a subject's underlying condition or not expected as a risk of the study; therefore, not included in the investigator's brochure, protocol, or informed consent document),
   AND
2. Probably or definitely related to participation in the research,
   AND
3. Suggests that the research places subjects or others at a greater risk of harm (including physical, psychological, economic, or social harm) than was previously known or recognized. Note: According to OHRP, if the adverse event is serious, it would always suggest a greater risk of harm.

For purposes of this policy, UPIRSOs include unanticipated adverse device effects (UADEs) and death or serious injury related to a humanitarian use device (HUD).

UPIRSOs must be promptly reported to the UTSW HRPP within 5 working days of

study team awareness.

For research relying on a non-UT Southwestern IRB (external, central, or single IRB):

Investigators relying on an external IRB who are conducting research on behalf of UT Southwestern or its affiliates are responsible for submitting **LOCAL** UPIRSOs to the UT Southwestern IRB within 5 working days of study team awareness. Investigators must report to their relying IRB according to the relying IRB's policy. In addition, the external IRB's responses or determinations on these local events must be submitted to the UT Southwestern IRB within 10 working days of receipt.

Events NOT meeting UPIRSO criteria:

Events that do NOT meet UPIRSO criteria should be tracked, evaluated, summarized, and submitted to the UTSW HRPP/IRB at continuing review.

For more information on UTSW HRPP/IRB reportable event policy, see https://www.utsouthwestern.edu/research/hrpp/quality-assurance/

8.3.4.3 Reporting SAEs to Study Sponsors

Local serious adverse events (SAEs) for studies where SCCC DSMC is the DSMC of record require reporting to the DSMC coordinator within 2 working days of PI awareness, or as described in the protocol.

For all IST studies using a OncXerna Therapeutics Product: OncXerna Therapeutics requires that, within 24 hours of first awareness of event (or immediately if the event is fatal or life-threatening), the principal investigator will report to OncXerna Therapeutics by e-mail any Serious Adverse Event (SAE) that occurs during the SAE reporting period in a study subject assigned to receive the OncXerna Therapeutics product.
Contact information for SAE reporting to MedAssessment Pharmacovigilance and PPD medical monitors is as follows:

Safety Reporting Email (for submission of SAE related source documents): oncxernasafety@medassessment.com.

Additionally, any serious adverse event, considered by an investigator who is a qualified physician to be related to Merck product that is brought to the attention of the investigator at any time following consent through the end of the specified safety follow-up period or at any time outside of the time period specified in the previous paragraph also must be reported within 24 hours of first awareness of

event to Merck Global Safety. All subjects with serious adverse events must be followed up for outcome.

SAE reports and any other relevant safety information are to be forwarded to the Merck Global Safety facsimile number: +1-215-661-6229 A copy of all 15 Day Reports and Annual Progress Reports is submitted as required by FDA, European Union (EU), Pharmaceutical and Medical Devices agency (PMDA) or other local regulators. Investigators will cross reference this submission according to local regulations to the Merck Investigational Compound Number (IND, CSA, etc.) at the time of submission. Additionally investigators will submit a copy of these reports to Merck & Co., Inc. (Attn: Worldwide Product Safety; FAX 215-661-6229) at the time of submission to FDA.

8.4 STOPPING RULES

Simmons Comprehensive Cancer Center Data and Safety Monitoring Board (DSMB) will oversee this study and will determine if early stoppage of the trial is needed due to unacceptable rates of drug related serious adverse events.

8.5 STEPS TO DETERMINE IF AN ADVERSE EVENT REQUIRES EXPEDITED REPORTING

Step 1: Identify the type of adverse event using the NCI Common Terminology Criteria for Adverse Events (CTCAE v. 4.03).

Step 2: Grade the adverse event using the NCI CTCAE v. 4.03.

Step 3: Determine whether the adverse event is related to the protocol therapy Attribution categories are as follows:

- Definite – The AE *is clearly related* to the study treatment.
- Probable – The AE *is likely related* to the study treatment.
- Possible – The AE *may be related* to the study treatment.
- Unrelated – The AE *is clearly NOT related* to the study treatment.

Note: This includes all events that occur within 30 days of the last dose of protocol treatment. Any event that occurs more than 30 days after the last dose of treatment and is attributed (possibly, probably, or definitely) to the agent(s) must also be reported accordingly.

Step 4: Determine the prior experience of the adverse event.
Expected events are those that have been previously identified as resulting from administration of the agent. An adverse event is considered unexpected, for expedited reporting purposes only, when either the type of event or the severity of the event is not listed in:

- the current known adverse events listed in the Agent Information Section of this protocol;
- the drug package insert;
- the current Investigator’s Brochure

8.6 DEFINITION OF AN OVERDOSE FOR THIS PROTOCOL AND REPORTING OF OVERDOSE TO MERCK

For purposes of this trial, an overdose of pembrolizumab will be defined as any dose of 1,000 mg or greater (≥5 times the indicated dose). No specific information is available on the treatment of overdose of pembrolizumab. Appropriate supportive treatment should be provided if clinically indicated. In the event of overdose, the subject should be observed closely for signs of toxicity. Appropriate supportive treatment should be provided if clinically indicated.

If an adverse event(s) is associated with ("results from") the overdose of a Merck product, the adverse event(s) is reported as a serious adverse event, even if no other seriousness criteria are met. If a dose of Merck's product meeting the protocol definition of overdose is taken without any associated clinical symptoms or abnormal laboratory results, the overdose is reported as a non-serious Event of Clinical Interest (ECI), using the terminology "accidental or intentional overdose without adverse effect."

All reports of overdose with and without an adverse event must be reported within 24 hours to Merck Global Safety. (Attn: Worldwide Product Safety; FAX 215-661-6229-1220).

8.7 REPORTING OF PREGNANCY AND LACTATION TO MERCK

Although pregnancy and lactation are not considered adverse events, it is the responsibility of investigators or their designees to report any pregnancy or lactation in a subject (spontaneously reported to them) that occurs during the trial.

Pregnancies and lactations that occur after the consent form is signed but before treatment allocation/randomization must be reported by the investigator if they cause the subject to be excluded from the trial, or are the result of a protocol-specified intervention, including but not limited to washout or discontinuation of usual therapy, diet, placebo treatment or a procedure.

Pregnancies and lactations that occur from the time of treatment allocation/randomization through 120 days following cessation of Sponsor's product, or 30 days following cessation of treatment if the subject initiates new anticancer therapy, whichever is earlier, must be reported by the investigator. All reported pregnancies must be followed to the completion/termination of the pregnancy. Pregnancy, outcomes of spontaneous abortion, missed abortion, benign hydatidiform mole, blighted ovum, fetal death, intrauterine death, miscarriage and stillbirth must be reported as serious events (Important Medical Events). If the pregnancy continues to term, the outcome (health of infant) must also be reported.

Such events must be reported within 24 hours to Merck Global Safety. (Attn: Worldwide Product Safety; FAX 215-661-6229).

8.8 EVENTS OF CLINICAL INTEREST

Selected non-serious and serious adverse events are also known as Events of Clinical Interest (ECI) and must be reported within 24 hours to Merck Global Safety. (Attn: Worldwide Product Safety; FAX 215-661-6229).

For the time period beginning when the consent form is signed until treatment allocation/randomization, any ECI, or follow up to an ECI, that occurs to any subject must be reported within 24 hours to Merck Global Safety if it causes the subject to be excluded from the trial, or is the result of a protocol-specified intervention, including but not limited to washout or discontinuation of usual therapy, diet, placebo treatment or a procedure.

For the time period beginning at treatment allocation/randomization through 90 days following cessation of treatment, or 30 days following cessation of treatment if the subject initiates new anticancer therapy, whichever is earlier, any ECI, or follow up to an ECI, whether or not related to Merck product, must be reported within 24 hours to Merck Global Safety.

Events of clinical interest for this trial include:

1. An overdose of Merck product, as defined in Section 8.6 - Definition of an Overdose for This Protocol and Reporting of Overdose to the Sponsor, that is not associated with clinical symptoms or abnormal laboratory results.
2. An elevated AST or ALT lab value as referenced in Section 5.8

9.0 CORRELATIVES/SPECIAL STUDIES

Pathology Correlative Studies

A major obstacle to molecularly targeted chemotherapeutic agents has been obtaining relevant tumor tissue to measure the agents' biologic effects on target molecules or cellular pathways within the tumor. The utilization of sequential tumor biopsies to obtain tissue following treatment is well established in breast and colorectal carcinoma. (35). Liver biopsies of HCC have been shown to be safe. A large, multi-institutional trial where patients with advanced chronic Hepatitis C underwent sequential liver biopsies demonstrated 16 cases (0.6%) of bleeding following 2740 liver biopsies. There were no deaths from periprocedural bleeding and a platelet count under 60000 was identified as a risk factor. (38) Two large published series of core needle biopsy of HCC as a diagnostic measure report bleeding events in less than 1% of patients with no reported mortalities. In both series capsular location of HCC was associated with increased incidence of bleeding event. (39, 40) In this study all patients will potentially undergo up to two image-guided (either CT or Ultrasound) core needle biopsy of a single site of HCC at baseline (for clinical care) and between three and four weeks after the initiation of study drug (research only). The biopsy between three and four weeks following study drug initiation is not mandatory to initiate or continue with therapy and is subject to a separate informed consent. The baseline biopsy is needed to confirm HCC as an eligibility criterion. If archival formalin fixed paraffin embedded tissue is available for HCC status analysis, a pre-treatment biopsy will not be required. To increase patient safety the following conditions must be met prior to liver biopsy:

- Platelet count ≥ 70,000;
- INR ≤ 1.8;
- Target lesion not subcapsular in nature as determined by the participating interventional radiology team;

The patient will sign the informed consent for participation in research liver biopsy prior to the procedure. The liver biopsy will be performed by radiologists and the ancillary staff of Department of Radiology following the standard clinical protocol, which includes obtaining a separate informed consent for the procedure itself. The core samples will be provided to the Department of Pathology

for histological confirmation in addition to the pathology laboratory. If a core biopsy is performed as a part of the patient's clinical care, extra cores will be obtained for surgical oncology laboratory, in addition to the cores necessary for clinical care. All cores will be obtained through the same needle to avoid the need of additional punctures and to minimize associated risks.

Tumor tissue may be used for RNA, DNA, and/or protein testing.

10.0 STUDY MANAGEMENT

10.1 CONFLICT OF INTEREST

Any investigator who has a conflict of interest with this study (patent ownership, royalties, or financial gain greater than the minimum allowable by their institution, etc.) must have the conflict reviewed by the UTSW COI Committee and IRB according to UTSW Policy on

Conflicts of Interest. All investigators will follow the University conflict of interest policy.

10.2 INSTITUTIONAL REVIEW BOARD (IRB) APPROVAL AND CONSENT

It is expected that the IRB will have the proper representation and function in accordance with federally mandated regulations. The IRB must approve the consent form and protocol.

In obtaining and documenting informed consent, the investigator should comply with the applicable regulatory requirement(s), and should adhere to Good Clinical Practice (GCP) and to ethical principles that have their origin in the Declaration of Helsinki.

Before recruitment and enrollment onto this study, the subject will be given a full explanation of the study and will be given the opportunity to review the consent form. Each consent form must include all the relevant elements currently required by the FDA Regulations and local or state regulations. Once this essential information has been provided to the subject and the investigator is assured that the subject understands the implications of participating in the study, the subject will be asked to give consent to participate in the study by signing an IRB-approved consent form.

Prior to a patient's participation in the trial, the written informed consent form should be signed and personally dated by the subject and by the person who conducted the informed consent discussion.

10.3 RECRUITMENT PLAN

The study will be open to all patients seen at the UT Southwestern Medical Center

(including Parkland Health & Hospital System, University Hospital-Clements and Zale, and the Harold C. Simmons Comprehensive Cancer Center) and participating subsites.

Patients will be identified from surgical, hepatology, gastroenterology, radiology and medical oncology clinics for treatment of their disease. After a discussion of the patient's disease and a formulation of the initial treatment plan, the physician-investigator will describe the study to the patient. The protocol will be discussed in a private clinic room or office. Details including the risks and obligations of the subjects will be explained. For non-English speaking patients, an independent translator will be available to communicate

the details of the protocol. A research coordinator will be available either in the clinic or by phone to answer any additional questions.

Upon completion of the informed consent form and confirmation of protocol eligibility, the Clinical Research Office (CRO) at the Harold C. Simmons Comprehensive Cancer Center will be notified of the new enrollment. All patients will be entered into the Velos Clinical Trials Management System for ongoing monitoring. All data collected will be entered into the password protected REDCap electronic data capture system.

The investigators take due notice of the NIH policy concerning inclusion of women and minorities in clinical research populations. There will be no limitation to access with regard to race or gender. Patients will be required to read, agree to and sign an IRB-approved informed consent form prior to registration on this trial. The registration procedure will be conducted as described above. Patients will not receive payment for their participation on this study.

10.4 STUDY SUBJECT IDENTIFICATION NUMBER

All subjects will be assigned a study number that is not linked to their personal identifiers to prevent loss of confidentiality. The number will be the Site Number, 01, 02, etc. followed by a sequential number starting with number 001. For example, the first subject enrolled from site # 01 will be assigned study subject number 01-001; the next will be 01-002, and so forth. For this protocol, current sites are: 01 – UTSW and 02 – PHHS. Any additional sites added to this protocol will be numbered sequentially…03, 04, etc. This number will be assigned by the UTSW - Clinical Research Manager (CRM) or delegate upon confirmation of subject eligibility during the subject registration process.

10.5 SUBJECT REGISTRATION

Following completion of baseline assessments and confirmation of subject eligibility, patients will be assigned a subject identification number by the UTSW - CRM or delegate and registered into Velos, the UTSW Clinical Trial

Management System (CTMS). Subject registration will be confirmed (within 24 hours) once the following documents have been received by the UTSWCRM or delegate.

- Signed copy of the Informed Consent signature page;
- Inclusion/Exclusion worksheet signed by the coordinator and treating physician;

All subjects must be registered with the UTSW - CRM before enrollment to study. To register a subject, email the above supporting documents to the UTSW – CRM: Ellen Siglinsky, ellen.siglinsky@utsouthwestern.edu or call 214-648-7029, Monday through Friday, 9:00a.m. - 5:00p.m. CST.

10.6 PROTECTION OF HUMAN SUBJECTS

Participation in this trial is voluntary. All patients will be required to sign a statement of informed consent, which must conform to IRB guidelines.

Inclusion of Women and Minorities: Patients of all races, both male and female, will be accepted into the protocol.

Exclusion of Lactating or Pregnant Women: Children have been excluded from this study. Hepatocellular carcinoma is an adult cancer. Thus, the relevance of this drug to the pediatric population has not been established. Lactating and pregnant women are also excluded because of potential anti-proliferative effects of therapy that may be harmful to the developing fetus or nursing infant.

Benefits: It is possible that this treatment will result in shrinkage of hepatocellular carcinoma or in a stabilization of an otherwise progressing disease. It is not known, of course, whether these or any other favorable events will occur. It is not known whether this treatment will affect overall survival of the patients.

Costs: The patient will be responsible for the costs of standard medical care.

Pembrolizumab and bavituximab will be supplied to patients without costs from Merck and OncXerna Therapeutics. Patients will not be responsible for the costs of tissue or serum procurement (including image guided biopsies) obtained for research purposes.

Incentives: No incentives will be offered to patients/subjects for participation in this study.

Alternatives: For patients with advanced hepatocellular carcinoma, alternative treatments may include other chemotherapy regimens or loco-regional therapies including transarterial chemoembolization and radiofrequency ablation. At present, no specific treatment approach is considered standard of care for the disease. Patients may be eligible for other investigational studies.

Confidentiality: Every effort will be made to maintain patient confidentiality. Research and hospital records are confidential. Patient's name or any personally identifying information will not be used in reports or publications resulting from this study. The Food and Drug Administration or other authorized agencies (i.e., qualified monitors from UT Southwestern Medical Center, the NCI, etc.), may review patients records and pathology slides, as required.

10.7 DATA MANAGEMENT AND MONITORING/AUDITING

REDCap is the UTSW SCCC institutional choice for the electronic data capture of case report forms for this and all SCCC Investigator Initiated Trials. REDCap will be used for electronic case report forms in accordance with Simmons Comprehensive Cancer Center requirements.

Trial monitoring will be conducted according to the study specific monitoring plan. For guidance on creating a monitoring plan, refer to the UTSW SCCC IIT Management Manual.

Toxicity and dose escalation reviews will be performed in real-time by the GI DOT and DSMC. These reviews will be documented by distributing reports of the findings to the members of the GI DOT and investigators.

The UTSW Simmons Comprehensive Cancer Center (SCCC) Data Safety Monitoring Committee (DSMC) is responsible for monitoring data quality and patient safety for all UTSW SCCC clinical trials. As part of that responsibility, the DSMC reviews all local serious adverse events and unanticipated problems in real time as they are reported and reviews adverse events on a quarterly basis. The quality assurance activity for the Clinical Research Office provides for periodic auditing of clinical research documents to ensure data integrity and regulatory compliance. A copy of the DSMC plan is available upon request.

The SCCC DSMC meets quarterly and conducts annual comprehensive reviews of ongoing clinical trials, for which it serves as the DSMC of record. The QAC works as part of the DSMC to conduct regular audits based on the level of risk. Audit findings are reviewed at the next available DSMC meeting. In this way, frequency of DSMC monitoring is dependent upon the level of risk. Risk level is determined by the DSMC Chairman and a number of factors such as the phase of the study; the type of investigational agent, device or intervention being studied; and monitoring required to ensure the safety of study subjects based on the associated risks of the study. Protocol-specific DSMC plans must be consistent with these principles. Safety is monitored in real-time as adverse events are submitted to the DSMC and IRB with attributions.

.

10.8 ADHERENCE TO THE PROTOCOL

Except for an emergency situation, in which proper care for the protection, safety, and well-being of the study subject requires alternative treatment, the study shall be conducted exactly as described in the approved protocol.

10.8.1 Exceptions

Exceptions (also called single-subject exceptions or single-subject waivers) include any departure from IRB-approved research that is *not due to an emergency* and is:

- intentional on part of the investigator; or
- in the investigator’s control; or
- not intended as a systemic change (e.g., single-subject exceptions to eligibility [inclusion/exclusion] criteria)
- **Reporting requirement***: Exceptions are non-emergency deviations that require ***prospective*** IRB approval before being implemented. Call the IRB if your request is urgent. If IRB approval is not obtained beforehand, this constitutes a major deviation. For eligibility waivers, studies which utilize the SCCC-DSMC as the DSMC of record must also obtain approval from the DSMC prior to submitting to IRB for approval.

10.8.2 Emergency Deviations: include any departure from IRB-approved research that is necessary to:

- **avoid immediate apparent harm, or**
- **protect the life or physical well-being of subjects or others**

 Reporting requirement*: Emergency deviations must be promptly reported to the IRB within 5 working days of occurrence.

10.8.3 Serious Noncompliance (formerly called major deviations or violations): include any departure from IRB-approved research that:

- Increase risk of harm to subjects; and/or
  adversely affects the rights, safety, or welfare of subjects (any of which may also be an unanticipated problem); and/or
- adversely affects the integrity of the data and research (i.e., substantially compromises the integrity, reliability, or validity of the research)

 Reporting requirement*: Serious Noncompliance must be promptly reported to the IRB within 5 working days of discovery.

10.8.4 Continuing Noncompliance: includes a pattern of repeated noncompliance (in or more protocols simultaneously, or over a period of time) which continues after initial discovery, including inadequate efforts to take or implement corrective or preventive action within a reasonable time frame.

 Reporting requirement*: Continuing Noncompliance must be promptly reported to the IRB within 5 working days of discovery.

10.8.5 Noncompliance (that is neither serious nor continuing; formerly called minor deviations) any departure from IRB-approved research that:

- **Does not meet the definition of serious noncompliance or continuing noncompliance**

 Reporting requirement*: Noncompliance that is neither serious nor continuing should be tracked and summarized the next IRB continuing review, or the notice of study closure- whichever comes first..

*Reporting Requirements reflect UTSW HRPP/IRB guidelines; participating sites should follow the reporting guidelines for their IRB of record

10.9 AMENDMENTS TO THE PROTOCOL

Should amendments to the protocol be required, the amendments will be originated and documented by the Principal Investigator. A summary of changes document outlining proposed changes as well as rationale for changes, when appropriate, is highly recommended. When an amendment to the protocol substantially alters the study design or the potential risk to the patient, a revised consent form might be required.

The written amendment, and if required the amended consent form, must be sent to the IRB for approval prior to implementation.

10.10 RECORD RETENTION

Study documentation includes all Case Report Forms, data correction forms or queries, source documents, Sponsor-Investigator correspondence, monitoring logs/letters, and regulatory documents (e.g., protocol and amendments, IRB correspondence and approval, signed patient consent forms).

Source documents include all recordings of observations or notations of clinical activities and all reports and records necessary for the evaluation and reconstruction of the clinical research study.

Government agency regulations and directives require that the study investigator retain all study documentation pertaining to the conduct of a clinical trial. In the case of a study with a drug seeking regulatory approval and marketing, these documents shall be retained for at least two years after the last approval of marketing application in an International Conference on Harmonization (ICH) region. In all other cases, study documents should be kept on file until three years after the completion and final study report of this investigational study.

10.11 OBLIGATIONS OF INVESTIGATORS

The Principal Investigator is responsible for the conduct of the clinical trial at the site in accordance with Title 21 of the Code of Federal Regulations and/or the Declaration of Helsinki. The Principal Investigator is responsible for personally overseeing the treatment of all study patients. The Principal Investigator must assure that all study site personnel, including sub-investigators and other study staff members, adhere to the study protocol and all FDA/GCP/NCI regulations and guidelines regarding clinical trials both during and after study completion.

The Principal Investigator at each institution or site will be responsible for assuring that all the required data will be collected and entered onto the Case Report Forms. Periodically, monitoring visits may be conducted and the Principal Investigator will provide access to his/her original records to permit verification of proper entry of data. At the completion of the study, all case report forms will be reviewed by the Principal Investigator and will require his/her final signature to verify the accuracy of the data.

11.0 REFERENCES

1. Parkin DM, Bray F, Ferlay J, et al. Estimating the world cancer burden: Globocan 2000. Int J Cancer 2001; 94(2):153-6.
2. Altekruse SF, McGlynn KA, Reichman ME. Hepatocellular carcinoma incidence, mortality, and survival trends in the United States from 1975 to 2005. J Clin Oncol 2009; 27(9):148591.
3. El-Serag HB, Mason AC. Rising incidence of hepatocellular carcinoma in the United States. N Engl J Med 1999; 340(10):745-50.

4. El-Serag HB. Hepatocellular carcinoma: recent trends in the United States. Gastroenterology 2004; 127(5 Suppl 1):S27-34.
5. Chen MS, Li JQ, Zheng Y, et al. A prospective randomized trial comparing percutaneous local ablative therapy and partial hepatectomy for small hepatocellular carcinoma. Ann Surg 2006; 243(3):321-8.
6. Shiina S, Teratani T, Obi S, et al. A randomized controlled trial of radiofrequency ablation with ethanol injection for small hepatocellular carcinoma. Gastroenterology 2005; 129(1):122-30.
7. Llovet JM, Real MI, Montana X, et al. Arterial embolisation or chemoembolisation versus symptomatic treatment in patients with unresectable hepatocellular carcinoma: a randomised controlled trial. Lancet 2002; 359(9319):1734-9.
8. Lo CM, Ngan H, Tso WK, et al. Randomized controlled trial of transarterial lipiodol chemoembolization for unresectable hepatocellular carcinoma. Hepatology 2002; 35(5):1164-71.
9. Boige V, Taieb J, Hebbar M, et al. Irinotecan as first-line chemotherapy in patients with advanced hepatocellular carcinoma: a multicenter phase II study with dose adjustment according to baseline serum bilirubin level. Eur J Cancer 2006; 42(4):456-9.
10. Guan Z, Wang Y, Maoleekoonpairoj S, et al. Prospective randomised phase II study of gemcitabine at standard or fixed dose rate schedule in unresectable hepatocellular carcinoma. Br J Cancer 2003; 89(10):1865-9.
11. Gish RG, Porta C, Lazar L, et al. Phase III randomized controlled trial comparing the survival of patients with unresectable hepatocellular carcinoma treated with nolatrexed or doxorubicin. J Clin Oncol 2007; 25(21):3069-75.
12. Yeo W, Mok TS, Zee B, et al. A randomized phase III study of doxorubicin versus cisplatin/interferon alpha-2B/ doxorubicin/fluorouracil (PIAF) combination chemotherapy for unresectable hepatocellular carcinoma. J Natl Cancer Inst 2005;97(20):1532-8.
13. Wilhelm SM, Carter C, Tang L, et al. BAY 43-9006 exhibits broad spectrum oral antitumor activity and targets the RAF/MEK/ERK pathway and receptor tyrosine kinases involved in tumor progression and angiogenesis. Cancer Res 2004; 64(19):7099-109.
14. Llovet JM, Ricci S, Mazzaferro V, et al. Sorafenib in advanced hepatocellular carcinoma. N Engl J Med 2008; 359(4):378-90.
15. Lencioni R, Kudo M, Ye SL, et al: GIDEON (Global Investigation of therapeutic DEcisions in hepatocellular carcinoma and Of its treatment with sorafeNib): second interim analysis. Int J Clin Pract 2014;68:609-17.
16. Llovet JM, Hernandez-Gea V: Hepatocellular carcinoma: reasons for phase III failure and novel perspectives on trial design. Clin Cancer Res 2014; 20:2072-9.
17 Stauffer JK, Scarzello AJ, Jiang Q, et al: Chronic inflammation, immune escape, and oncogenesis in the liver: a unique neighborhood for novel intersections. Hepatology 2012; 56:1567-74.
18. Greten TF, Wang XW, Korangy F: Current concepts of immune based treatments for patients with HCC: from basic science to novel treatment approaches. Gut 2015;64:842-8.
19. Hato T, Goyal L, Greten TF, et al: Immune checkpoint blockade in hepatocellular carcinoma: current progress and future directions. Hepatology 2014;60:1776-82.
20. Oquinena S, Guillen-Grima F, Inarrairaegui M, et al: Spontaneous regression of hepatocellular carcinoma: a systematic review. Eur J Gastroenterol Hepatol 2009;21:254-7.

21. Huz JI, Melis M, Sarpel U: Spontaneous regression of hepatocellular carcinoma is most often associated with tumour hypoxia or a systemic inflammatory response. HPB (Oxford) 2012; 14:500-5.
22. Unitt E, Marshall A, Gelson W, et al: Tumour lymphocytic infiltrate and recurrence of hepatocellular carcinoma following liver transplantation. J Hepatol 2006;45:246-53.
23. Takayama T, Sekine T, Makuuchi M, et al: Adoptive immunotherapy to lower postsurgical recurrence rates of hepatocellular carcinoma: a randomised trial. Lancet 2000;356:802-7.
24. Breous E, Thimme R: Potential of immunotherapy for hepatocellular carcinoma. J Hepatol 2011;54:830-4.
25. Sangro B, Gomez-Martin C, de la Mata M, et al: A clinical trial of CTLA-4 blockade with tremelimumab in patients with hepatocellular carcinoma and chronic hepatitis C. J Hepatol 2013;59:81-8.
26. Wu P, Wu D, Li L, et al: PD-L1 and Survival in Solid Tumors: A Meta-Analysis. PLoS One 2015; 10:e0131403.
27. El-Khoueiry AN, Melero I, Crocenzi TS, et al: Phase I/II safety and antitumor activity of nivolumab in patients with advanced hepatocellular carcinoma (HCC). J Clin Oncol 2015; 33, abtr LBA 101.
28. Robert C, Schachter J, Long GV, et al: Pembrolizumab versus Ipilimumab in Advanced Melanoma. N Engl J Med 2015; 372:2521-32.
29. Garon EB, Rizvi NA, Hui R, et al: Pembrolizumab for the treatment of non-small-cell lung cancer. N Engl J Med 2015; 372:2018-28.
30. Yopp AC, Singal AG, Arriaga Y, et al: A phase II study of bavituximab and sorafenib in advanced hepatocellular carcinoma (HCC). J Clinic Oncol 2015;33,suppl 3, abstr 345.
31. Cheng X, Li L, Thorpe PE, et al: Antibody-Mediated Blockade of Phosphatidylserine Enhances the Antitumor Effect of Sorafenib in Hepatocellular Carcinomas Xenografts. Ann Surg Oncol, 2016
32. Gray MJ, Gong, J, Hatch MMS, et al: Phosphatidylserine-targeting antibodies augment the anti-tumorigenic activity of anti-PD-1 therapy by enhancing immune activation and downregulating pro-oncogenic factors induced by T-cell checkpoint inhibiton in murine triplenegative breast cancers. Breast Cancer Research 2016; doi 10.1186/s 13058-016-0708-2
33. Freimark B, Gong J, Ye D, et al: Antibody mediated phosphatidylserine blockade enhances the antitumor responses to CTLA-4 and PD-1 antibodies in melanoma. Cancer Immunol Res 2016;4(6):531-40.
34. Hodi FS, Hwu WJ, Kefford R, et al: Evaluation of Immune-Related Response Criteria and RECIST v1.1 in Patients With Advanced Melanoma Treated With Pembrolizumab. J Clin Oncol, 2016
35. Thomas MB, Jaffe D, Choti MM, et al: Hepatocellular carcinoma: consensus recommendations of the National Cancer Institute Clinical Trials Planning Meeting. J Clin Oncol 2010; 28:3994-4005.
36. Case, LD and Morgan, TM. Design of phase II cancer trials evaluating survival probabilities. BMC Medical Research Methodology 2003;3-6.
37. Dowlati A, Haaga J, Remick SC, et al. Sequential tumor biopsies in early phase clinical trial of anticancer agents for pharmacodynamic evaluation. Cancer Res 2001;7(10);2971-6.

38. Seeff LB, Everson GT, Morgan TR, et al. Complication rate of percutaneous liver biopsies among persons with advanced chronic liver disease in the HALT-C trial. Am J Gastro doi:10.1016lj.cgh.2010.03.025.
39. Giorgio A, Tarantino L, de Stefano, et al. Complications after interventional sonography of focal liver lesions: a 22-year single-center experience. J Ultrasound Med 2003;22:193-205.
40. Caturelli E, Solmi L, Anti M, et al. Ultrasound guided fine needle biopsy of early hepatocellular carcinoma complicating liver cirrhosis: a multicentre study. Gut 2004;53:135662.
41. El-Khoueiry AB, Sangro B, Yau T, et al. Nivolumab in patients with advanced hepatocellular carcinoma (CheckMate 040): an open-label, non-comparative, phase 1/2 dose escalation and expansion trial. Lancet 2017;389:2492-502.

12.0 APPENDICES

Appendix A: ECOG Performance Status

Grade	ECOG
0	Fully active, able to carry on all pre-disease performance without restriction
1	Restricted in physically strenuous activity but ambulatory and able to carry out work of a light or sedentary nature, i.e., light house work, office work
2	Ambulatory and capable of all self-care but unable to carry out any work activities. Up and about more than 50% of waking hours.
3	Capable of only limited self-care, confined to be or chair more than 50% of waking hours.
4	Completely disabled. Cannot carry on any self-care. Totally confined to bed or chair.
5	Dead

Appendix B: Child-Pugh Score

Measure	1 point	2 points	3 points
Total bilirubin (mg/dl)	<2.0	2.0-3.0	>3.0
Serum albumin (g/l)	>3.5	2.8-3.5	<3.5
INR	<1.7	1.7-2.2	>2.2
Ascites	None	Mild (based on clinical findings)	Severe (based on clinical findings)
Hepatic Encephalopathy	None	Controlled Medically	Refractory

5-6 points Child-Pugh A
7-9 points Child-Pugh B
10-15 points Child-Pugh C

Appendix C: NYHA Classification

Class	Patient Symptoms
I	No limitation of physical activity. Ordinary physical activity does not cause undue fatigue, palpitation, or dyspnea (shortness of breath).
II	Slight limitation of physical activity. Comfortable at rest, but ordinary physical activity results in fatigue, palpitation, or dyspnea.
III	Marked limitation of physical activity. Comfortable at rest, but less than ordinary activity causes fatigue, palpitation, or dyspnea.
IV	Unable to carry out any physical activity without discomfort. Symptoms of cardiac insufficiency at rest. If any physical activity is undertaken, discomfort is increased
