# Supplementary material for: The phosphatidylserine targeting antibody bavituximab plus pembrolizumab in unresectable hepatocellular carcinoma: a phase 2 trial
Source: Nat Commun. 2024 Mar 11;15:2178. doi: 10.1038/s41467-024-46542-y (PMC10928173; doi:10.1038/s41467-024-46542-y)
Supplement: Supplementary file 3 — Description of Additional Supplementary Files [file 41467_2024_46542_MOESM3_ESM.pdf]

Description of Additional Supplementary Files

Supplementary Data 1. The abundance of proteins in tumor and adjacent liver in tumor, immune cell, and stroma compartments were quantified using the NanoString GeoMx assay and normalized to house keeping genes. Each column denotes a region of interest of an individual patient of a particular tissue segment (cancer cell, bile duct, immune cell, or stroma).
