# Supplementary material for: The phosphatidylserine targeting antibody bavituximab plus pembrolizumab in unresectable hepatocellular carcinoma: a phase 2 trial
Source: Nat Commun. 2024 Mar 11;15:2178. doi: 10.1038/s41467-024-46542-y (PMC10928173; doi:10.1038/s41467-024-46542-y)
Supplement: Supplementary file 5 — Reporting Summary [file 41467_2024_46542_MOESM5_ESM.pdf]

nature portfolio

Corresponding author(s): David Hsiehchen

Last updated by author(s): Jun 7, 2023

Reporting Summary

Nature Portfolio wishes to improve the reproducibility of the work that we publish. This form provides structure for consistency and transparency in reporting. For further information on Nature Portfolio policies, see our Editorial Policies and the Editorial Policy Checklist.

Statistics

For all statistical analyses, confirm that the following items are present in the figure legend, table legend, main text, or Methods section.

n/a	Confirmed	
☐	☒	The exact sample size (*n*) for each experimental group/condition, given as a discrete number and unit of measurement
☐	☒	A statement on whether measurements were taken from distinct samples or whether the same sample was measured repeatedly
☐	☒	The statistical test(s) used AND whether they are one- or two-sided<br>*Only common tests should be described solely by name; describe more complex techniques in the Methods section.*
☒	☐	A description of all covariates tested
☐	☒	A description of any assumptions or corrections, such as tests of normality and adjustment for multiple comparisons
☐	☒	A full description of the statistical parameters including central tendency (e.g. means) or other basic estimates (e.g. regression coefficient) AND variation (e.g. standard deviation) or associated estimates of uncertainty (e.g. confidence intervals)
☐	☒	For null hypothesis testing, the test statistic (e.g. *F*, *t*, *r*) with confidence intervals, effect sizes, degrees of freedom and *P* value noted<br>*Give P values as exact values whenever suitable.*
☒	☐	For Bayesian analysis, information on the choice of priors and Markov chain Monte Carlo settings
☒	☐	For hierarchical and complex designs, identification of the appropriate level for tests and full reporting of outcomes
☒	☐	Estimates of effect sizes (e.g. Cohen's *d*, Pearson's *r*), indicating how they were calculated

Our web collection on statistics for biologists contains articles on many of the points above.

Software and code

Policy information about availability of computer code

Data collection	No software was used for data collection.
Data analysis	No custom code was used. Analyses were performed using SPSS version 24.

For manuscripts utilizing custom algorithms or software that are central to the research but not yet described in published literature, software must be made available to editors and reviewers. We strongly encourage code deposition in a community repository (e.g. GitHub). See the Nature Portfolio guidelines for submitting code & software for further information.

Data

Policy information about availability of data

All manuscripts must include a data availability statement. This statement should provide the following information, where applicable:

- Accession codes, unique identifiers, or web links for publicly available datasets
- A description of any restrictions on data availability
- For clinical datasets or third party data, please ensure that the statement adheres to our policy

Individual de-identified clinical data may be requested through the corresponding author David Hsiehchen (David.hsieh@utsouthwestern.edu), which will require the approval of the institutional review board. Written requests that include data required and study purpose will be responded to within 6 weeks. Spatial profiling data are publicly available via the NCBI Gene Expression Omnibus database (accession number: GSE242154 [https://www.ncbi.nlm.nih.gov/geo/query/acc.cgi?acc=GSE242154]). Raw sequencing data from genomic and RNA signature analyses are protected by privacy laws and not publicly accessible because the study consent did not cover deposition of patient genetic data. Remaining data are available in the manuscript and Supplementary Information. Source data are provided

with this paper.

Research involving human participants, their data, or biological material

Policy information about studies with human participants or human data. See also policy information about sex, gender (identity/presentation), and sexual orientation and race, ethnicity and racism.

Reporting on sex and gender	Self-reported sex was used in subset analyses to assess the relationship between demographics and outcomes. Gender was not analyzed.
Reporting on race, ethnicity, or other socially relevant groupings	Race was used in subset analyses to assess the relationship between demographics and outcomes. No other social groupings were assessed.
Population characteristics	Detailed demographics and other patient characteristics are shown in Supplementary Table 1. Patients were eligible if they were 18 years or older and had a histologically confirmed diagnosis of HCC (excluding fibrolamellar, sarcomatoid, and combined subtypes), locally advanced or metastatic disease not amenable to surgical resection, transplantation, or locoregional therapies. Enrollment was limited to adults 18 and older. No patients were excluded based on sex/gender, race, ethnicity, or other demographic. Out of 28 evaluable patients, 24 were male, 14 were black patients, and 2 were Hispanic patients. These demographics are consistent with the demographics of patients seen at enrolling clinical sites.
Recruitment	Patients meeting all inclusion and exclusion criteria of the trial were screened at a academic cancer center and a county safety net hospital. No other specific recruiting procedures were used to enroll patients.
Ethics oversight	This study received approval from the University of Texas Southwestern Institutional Review Board.

Note that full information on the approval of the study protocol must also be provided in the manuscript.

Field-specific reporting

Please select the one below that is the best fit for your research. If you are not sure, read the appropriate sections before making your selection.

[x] Life sciences [ ] Behavioural & social sciences [ ] Ecological, evolutionary & environmental sciences

For a reference copy of the document with all sections, see nature.com/documents/nr-reporting-summary-flat.pdf

Life sciences study design

All studies must disclose on these points even when the disclosure is negative.

Sample size	We used minimax two-stage design method to compare the ORR of 0.35 (alternative hypothesis) for the treatment cohort versus 0.15 (null hypothesis) for the historical control. In the first stage 15 patients will be accrued. If there are 3 or more responses then 13 additional patients will be accrued for a total of 28 patients. The null hypothesis will be rejected if 8 or more responses are observed in 28 patients. This design yields a type I error rate of 0.05 and power of 0.80 when the true response rate is 0.35%.
Data exclusions	No data was excluded.
Replication	This was a single clinical trial.
Randomization	As a single-arm study, no randomization was performed.
Blinding	As an open-label study, there was no blinding.

Reporting for specific materials, systems and methods

We require information from authors about some types of materials, experimental systems and methods used in many studies. Here, indicate whether each material, system or method listed is relevant to your study. If you are not sure if a list item applies to your research, read the appropriate section before selecting a response.

Materials & experimental systems

n/a	Involved in the study
☐	☒ Antibodies
☒	☐ Eukaryotic cell lines
☒	☐ Palaeontology and archaeology
☒	☐ Animals and other organisms
☐	☒ Clinical data
☒	☐ Dual use research of concern
☒	☐ Plants

Methods

n/a	Involved in the study
☒	☐ ChIP-seq
☒	☐ Flow cytometry
☒	☐ MRI-based neuroimaging

Antibodies

Antibodies used	anti-SMA (Invitrogen, Catalog No. 50-9760-82, 1:40), anti-CD45 (Novus Biologicals, Catalog No. NBP1–44763AF594, 1:40), and anti-pan-cytokeratin (Pan-CK; GeoMx Solid Tumor TME Morphology Kit, NanoString, 1:40). Antibodies for 52 immuno-oncology-related proteins and three housekeeping/background proteins (GeoMx Immuno-Oncology Protein Panel, NanoString, 1:25).
Validation	All antibodies were validated by their commercial source:<br>1) https://www.thermofisher.com/antibody/product/Alpha-Smooth-Muscle-Actin-Antibody-clone-1A4-Monoclonal/50-9760-82<br>2) https://www.novusbio.com/products/cd45-antibody-em-05_nbp1-44763af594<br>3) https://nanostring.com/products/geomx-digital-spatial-profiler/geomx-morphology-markers<br>4) https://nanostring.com/products/geomx-digital-spatial-profiler/geomx-protein-assays/geomx-immuno-oncology-protein-panels/

Clinical data

Policy information about clinical studies

All manuscripts should comply with the ICMJEguidelines for publication of clinical research and a completedCONSORT checklist must be included with all submissions.

Clinical trial registration	NCT03519997
Study protocol	The study protocol has been included in the Supplementary Information.
Data collection	From 25 June 2018 to 2 March 2022, 42 patients were screened for eligibility at a National Cancer Institute-designated cancer center (UT Southwestern Harold C. Simmons Comprehensive Cancer Center) and a county safe-net hospital system (Parkland Health). 35 patients were enrolled, with 28 patients evaluable for the primary endpoint.
Outcomes	The primary endpoint was the confirmed overall response rate determined by the investigator using Response Evaluation Criteria in Solid Tumors (RECIST) 1.1 of all evaluable patients receiving at least one dose of pembrolizumab and bavituximab. Overall response rate was defined as number of patients with objective tumor responses (either CR or PR as best overall response) that were confirmed on imaging greater than or equal to 9 weeks after initial best response. Secondary endpoints included overall survival, progression free survival according to RECIST 1.1, duration of response, and safety.
